# Insights into the Variation in Bioactivities of Closely Related *Streptomyces* Strains from Marine Sediments of the Visayan Sea against ESKAPE and Ovarian Cancer

**DOI:** 10.3390/md19080441

**Published:** 2021-07-31

**Authors:** Edna M. Sabido, Chuckcris P. Tenebro, Dana Joanne Von L. Trono, Carmela Vannette B. Vicera, Sheeny Fane L. Leonida, Jose Jeffrey Wayne B. Maybay, Rikka Reyes-Salarda, Diana S. Amago, Angelica Marie V. Aguadera, May C. Octaviano, Jonel P. Saludes, Doralyn S. Dalisay

**Affiliations:** 1 Center for Natural Drug Discovery and Development (CND3), University of San Agustin, Iloilo City 5000, Philippines; ednasabido@usa.edu.ph (E.M.S.); sfleonida@usa.edu.ph (S.F.L.L.); jjmaybay@usa.edu.ph (J.J.W.B.M.); damago@usa.edu.ph (D.S.A.); aaguadera@usa.edu.ph (A.M.V.A.); moctaviano@usa.edu.ph (M.C.O.); 2 Center for Chemical Biology and Biotechnology (C2B2), University of San Agustin, Iloilo City 5000, Philippines; ctenebro@usa.edu.ph (C.P.T.); djvtrono@usa.edu.ph (D.J.V.L.T.); cvicera@usa.edu.ph (C.V.B.V.); rikkareyes@usa.edu.ph (R.R.-S.); 3 Department of Biology, College of Liberal Arts, Sciences, and Education, University of San Agustin, Iloilo City 5000, Philippines; 4 Department of Chemistry, College of Liberal Arts, Sciences, and Education, University of San Agustin, Iloilo City 5000, Philippines; 5 Tuklas Lunas Development Center, University of San Agustin, Iloilo City 5000, Philippines; 6 Balik Scientist Program, Department of Science and Technology, Philippine Council for Health Research and Development (PCHRD), Bicutan, Taguig City 1631, Philippines

**Keywords:** marine *Streptomyces* strains, antibiotics, marine sediments, chemodiversity, polyketide synthase, nonribosomal peptide synthetase, metabolomics, ESKAPE pathogens

## Abstract

Marine sediments host diverse actinomycetes that serve as a source of new natural products to combat infectious diseases and cancer. Here, we report the biodiversity, bioactivities against ESKAPE pathogens (*Enterococcus faecium*, *Staphylococcus aureus*, *Klebsiella pneumoniae*, *Acinetobacter baumannii*, *Pseudomonas aeruginosa*, and *Enterobacter* spp.) and ovarian cancer, and metabolites variation among culturable actinomycetes isolated from the marine sediments of Visayan Sea, Philippines. We identified 15 *Streptomyces* species based on a 16S rRNA gene sequence analysis. The crude extracts of 10 *Streptomyces* species have inhibited the growth of ESKAPE pathogens with minimum inhibitory concentration (MIC) values ranging from 0.312 mg/mL to 20 mg/mL depending on the strain and pathogens targeted. Additionally, ten crude extracts have antiproliferative activity against A2780 human ovarian carcinoma at 2 mg/mL. To highlight, we observed that four phylogenetically identical *Streptomyces albogriseolus* strains demonstrated variation in antibiotic and anticancer activities. These strains harbored type I and II polyketide synthase (PKS) and non-ribosomal synthetase (NRPS) genes in their genomes, implying that their bioactivity is independent of the polymerase chain reaction (PCR)-detected bio-synthetic gene clusters (BGCs) in this study. Metabolite profiling revealed that the taxonomically identical strains produced core and strain-specific metabolites. Thus, the chemical diversity among these strains influences the variation observed in their biological activities. This study expanded our knowledge on the potential of marine-derived *Streptomyces* residing from the unexplored regions of the Visayan Sea as a source of small molecules against ESKAPE pathogens and cancer. It also highlights that *Streptomyces* species strains produce unique strain-specific secondary metabolites; thus, offering new chemical space for natural product discovery.

## 1. Introduction

The dramatic increase in incidence of multidrug-resistant (MDR) bacterial infections has only recently stirred the revival of antimicrobial drug discovery programs because of the urgent need to combat emergent life-threatening bacterial infections from “ESKAPE” pathogens, namely, *Enterococcus faecium*, *Staphylococcus aureus*, *Klebsiella pneumoniae*, *Acinetobacter baumannii*, *Pseudomonas aeruginosa*, and *Enterobacter* spp. [1]. Measures and strategies are urgently needed worldwide to tackle drug resistance and promote research in antimicrobial drug discovery, including those from natural sources [2]. Interestingly, 41% of the new 32 antimicrobials reported since the year 2000 originated from actinomycetes. Thus, suggesting the vast potential of this group of microorganisms as a rich source of new antimicrobials [3]. Bacteria belonging to the family Actinomycetaceae are important natural sources of antimicrobial drugs [4,5,6]. The species of terrestrial bacteria belonging to this family, particularly the genus *Streptomyces,* have been demonstrated to prolifically produce diverse bioactive compounds currently sold in the market and prescribed for clinical use [4,7,8,9]. These free-living, saprophytic microorganisms could be found in diverse environments such as rain forests, lake and ocean sediments, estuaries, as well as deep ocean trenches, lake mud, beach sands, and sponges [5,10,11,12,13,14,15,16,17]. Actinomycetes are adaptive to their environment; thus, the secondary metabolites they produce are as variable as their location [18,19].

The production of secondary metabolites in Actinomycetes is driven by biosynthetic gene clusters (BGCs), a locally clustered group of two or more genes that encode a biosynthetic pathway [20,21]. Depending on the chemical entity of secondary metabolites, BGCs are differentiated according to different structural classes, including non-ribosomal peptide synthetases (NRPS), polyketide synthases (PKS), terpenes, and bacteriocins [22,23,24,25,26,27]. PKS and NRPS are popular BGC targets for natural product discovery. They are known to synthesize diverse chemical structures with potential medicine applications such as antibiotics, anticancer, and immunosuppressants [23,27,28,29,30,31]. The PKS small metabolites such as erythromycin (antibiotic) and rapamycin (immunosuppressant) are encoded in Type I polyketide synthase (type I PKS) [24]. Type II polyketide synthase (type II PKS) gene clusters form compounds through aromatization and cyclization such as tetracycline (antibiotic) and doxorubicin (anticancer) [26,27,29,30]. The PKS diversity evaluation often uses gene fragments of the type 1 PKS ketosynthase (KS) domain and the type II PKS KSα domain [32]. On the other hand, NRPs small metabolites such as antibiotics daptomycin [33] and vancomycin [34] are synthesized by nonribosomal peptide synthetase (NRPS). The NPRS adenylation (AD) domain is used for NRPs diversity evaluation [32,35,36].

Actinomycetes dwell in terrestrial soil, but evidence indicates that they are common in the marine ecosystem [17,18,37,38,39]. The strategy of using marine microorganisms as a source of new therapeutic agents is not unfeasible as it has been the focus of many recent investigations in other countries [5,13,20,40,41,42,43,44,45] and has led to the discovery of bioactive compounds with unique chemical entities. These include antitubercular sporalactam A and B [46] and dumulmycin [47], anticancer nahuoic acids [48,49] and kenalactams [50], and antibiotics bisanhydroaklavinone and 1-hydroxyaklavinone [11]. The conditions in the marine environment are extremely different from that of the terrestrial habitat: it is very demanding, nutrient deficient, competitive, and hostile and is inundated with high salinity and high pressure [18,38,39,51]. As a result, some metabolic changes occur in these bacteria that trigger the production of secondary metabolites that are distinct from those of their terrestrial counterparts [37,52]. Thus, there is a need to pursue actinomycetes from unexplored habitats such as the rich and biodiverse warm tropical marine environment.

The tropical marine environment of the Philippine archipelago is considered the epicenter of biodiversity and evolution as it lies in the Coral Triangle region [53,54]. The Philippines has a higher concentration of fish species per unit area than any Coral Triangle region [54]. Aside from marine fish and macro-organisms, microbes also dominate the marine ecosystem of the Philippine archipelago. However, its distribution, function, and biodiversity have mainly been largely undiscovered, poorly characterized taxonomically, and have not been harvested in culture to investigate natural products for potential pharmaceutical application. Recently, we isolated members of the phylum Actinobacteria, specifically the genus *Streptomyces,* from marine sediments of the Philippine archipelago. In our recent studies, one species of marine sediment-derived *Streptomyces* produces new anthracycline antibiotics that cause cell membrane damage in multidrug-resistant *Staphylococcus aureus* [11]. Further, a salt-tolerant new species of *Streptomyces* was isolated from marine sediments collected in the Islas de Gigantes group of islands in the northern part of Iloilo, Philippines, producing polycyclic aromatic polyketide angucycline glycosides fridamycin A and fridamycin D [12]. These angucycline antibiotics have activities against the multidrug-resistant *S. aureus* strain, harboring a drug-resistant gene SCCmec [12]. Our findings demonstrate that the marine sediment-derived *Streptomyces* from the Philippine archipelago is enriched with the potential to produce bioactive compounds and, thus, need further exploration.

In the present study, we continued investigating the biodiversity of cultivable streptomycetes sampled from marine sediments collected from various sites of the Islas de Gigantes group of islands in the Visayan Sea. In addition, we examined their antibacterial activity against ESKAPE pathogens and anticancer activity targeting ovarian cancer and their capability to produce bioactive secondary metabolites. The results revealed that marine sediments in the Islas de Gigantes group of islands are a reservoir of closely related *Streptomyces* species that displayed variation in activities against ESKAPE pathogens and ovarian cancer. To expand the metabolic potential of the isolates, we report the distribution of types I and II PKS and NRPS domains and how these BGC domains affect the bioactivities. Further, this study unveils the differences in metabolite composition of closely related strains of *Streptomyces* species, revealing that taxonomically identical species do not display matching metabolomic patterns. Notably, chemical diversity among these strains influences the variation observed in their biological activities. These findings offer new opportunities and a new chemical space for natural product discovery. Further, this study expanded our knowledge on the potential of marine-derived *Streptomyces* residing from the unexplored regions of the Visayan Sea as a source of small molecules against ESKAPE pathogens and cancer.

## 2. Results and Discussions

### 2.1. Cultivable Streptomycete Isolates in Marine Sediments from the Visayan Sea

**Figure 1:**
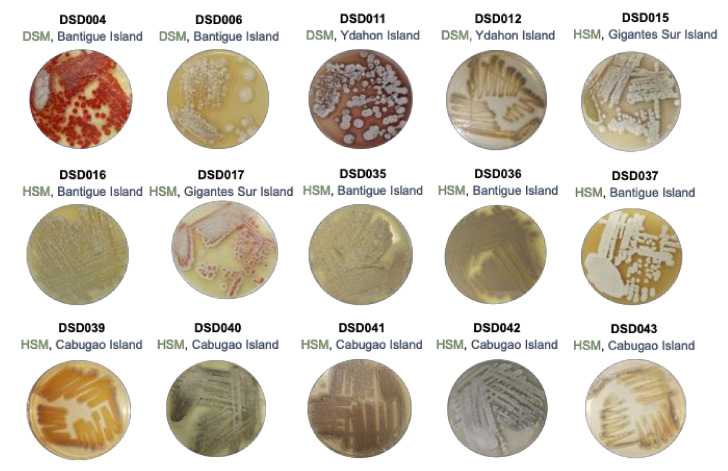
Colony phenotypes of streptomycete isolates in this study when grown in marine medium 1. The phylotype number (in black font), isolation method (in green font; DSM—Dry Stamp Method; HSM—Heat Shock Method), and collection site (in blue font) in Islas de Gigantes group of islands.

The Visayan Sea, where the Islas de Gigantes group of islands is located, is an extensive habitat for pelagic and demersal fish, thus, considered as one of the Philippines’ significant fishery resources. The topography of the Visayan Sea bottom is generally flat with infrequent minimum ascents and descents [54]. Certain areas in the Visayan Sea are studded with coral reefs and rocky shoals [54]. SCUBA collected a total of thirty (30) marine sediment samples at a depth of 20 to 30 m off the coast of Islas de Gigantes, Iloilo, Philippines. The sediments collected were classified as fine sand, silty sand, mud soil, or silty clay. Using the International *Streptomyces* Project Four (ISP4) medium in seawater as a selective medium for the isolation of streptomycetes, a total of fifteen (15) streptomycetes were identified based on the indicative characteristics of the genus *Streptomyces* such as colony morphologies, characteristics of aerial or substrate mycelium, and pigmentation. The isolates displayed morphological characteristics of streptomycetes such as leathery colonies, presence of aerial (spores), and substratum mycelium with diffusible pigments ranging from yellow, red, brown, to maroon (Figure 1). The streptomycete colonies were visible after 15 to 30 days of incubation, but some took as long as 60 days of incubation to appear in the ISP4 medium. The low number of isolated streptomycete isolates using ISP4 medium suggests that the use of other isolation media such as noble agar [11] and other isolation marine media with trehalose, raffinose, or mannitol as carbon source [13] are likely to result in the isolation of additional cultivable streptomycetes from the Visayan Seas. Among the isolates, 73% (11 isolates) were recovered by the heat shock method (HSM), while 27% (4 isolates) were recovered by the dry stamp method (DSM). The marine sediments from the Bantigue Island had the highest number of *Streptomyces* isolates (40%), followed by the Cabugao Island (33%) (5 isolates), and the marine sediments with the least number of streptomycetes recovered were from Ydahon, Antonia, and Gigantes Sur islands. The frequency in the number of isolated streptomycetes indicates that the marine sediments collected in the southeast islands of Islas de Gigantes, i.e., Bantigue and Cabugao islands (Supplemental Figure S1), are a good source of streptomycetes.

To determine whether the streptomycete isolates inhabiting the sediments of the Visayan Sea have an either terrestrial or marine origin, we grew the 15 isolates in MM1 medium prepared at different NaCl concentrations (0%, 3%, 5%, 7%, 10%, 12% 15%, and 20%). Results showed that all isolates grew abundantly (+++) from 0 to 5% NaCl concentration and moderate (++) to poor (+) growth between 7 and 12% NaCl concentration (Supplemental Table S1); thus, indicating that these isolates are salt-tolerant streptomycetes. Their ability to grow abundantly at 0% NaCl suggests that the marine sediment-derived *Streptomyces* in this study originated from the terrestrial environment and adapted well and evolved in the marine habitats in the Visayan Sea, as shown by their good growth behavior at 3 to 5% NaCl and growth tolerance at 12% NaCl. The members of Actinobacteria are typically present at densities on the order of 10^6^ to 10^9^ cells per gram of soil [55], with the genus *Streptomyces* dominating the soil population [4]. The spores are continually washed in the fresh or marine environment and are buried gradually in the seabed due to sedimenting particulate matter accumulation on the seafloor [39]. Due to the proximity of the collection sites in Islas de Gigantes Islands, there is a high probability that *Streptomyces* spores were washed into the sea and have evolved and adapted to the marine environment.

### 2.2. The Isolates from Marine Sediments of Visayan Sea Belong to Streptomyces Species

The BLAST analysis of the 16S rRNA gene sequences (1150–1490 bp) of marine sediment-derived streptomycetes isolated in the Visayas Sea near Islas de Gigantes revealed high sequence similarity values of 99–100% identity to *Streptomyces* species in the GenBank. The recovered *Streptomyces* species had a diversity index of 0.0492 suggesting low genetic diversity. The 16S rRNA gene sequences were deposited in GenBank with accession numbers: MZ323891, MZ323976, MT820508, MZ323983, MZ323974, MZ323997, MZ336041, MZ323985, MZ323984, MZ323989, MZ323987, MZ323957, MZ336042, MZ323972, and MZ323973.

**Figure 2:**
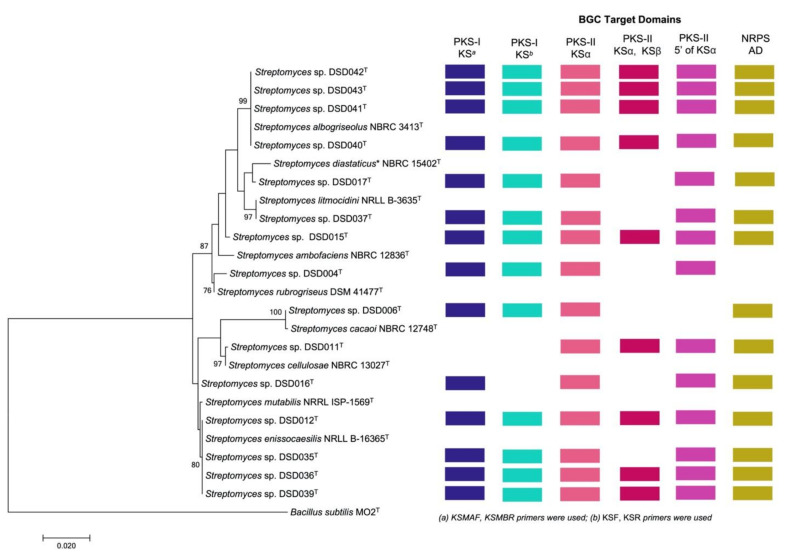
Phylogeny and biosynthetic gene cluster domain distribution in marine sediment-derived *Streptomyces* in this study. The phylogeny of *Streptomyces* isolates in this study with *Streptomyces* reference strains from the NCBI database are shown in maximum-likelihood phylogenetic tree based in the nearly completed 16S rRNA gene sequences. *Bacillus subtilis* MO2^T^ (AY553095.1) was used as an outgroup. Bootstrap values below 50% (based on 1000 replications) were not at the nodes. Colored boxes represent different biosynthetic gene cluster (BGC) target domains detected by PCR screening [12]. * *S. diastaticus* subsp. *ardesiacus*.

A phylogenetic tree was generated utilizing the MEGA 7 software and the level of confidence was calculated at a 1000 bootstrap value [56] to visualize the relatedness of the isolates and to their closely related *Streptomyces* species in the GenBank using the maximum-likelihood method (Figure 2). The level of confidence of ≥85% and ≥90% were considered in determining the relatedness of *Streptomyces* species in the phylogenetic tree while bootstrap values < 65% indicated a low confidence level in their evolutionary relatedness [36,57]. The *Streptomyces* species were clustered into seven clades in the phylogenetic tree. The first clade had a high degree of relatedness to *S. albogriseolus* NBRC 3413^T^ (99–100% similarity) and was supported by its high bootstrap value of 99%. Thus, the isolates belonging to this clad were designated as *S. albogriseolus* strains DSD040^T^ (1472 bp), DSD041^T^ (1479 bp), DSD042^T^ (1448 bp), and DSD043^T^ (1493 bp). Previous reports on *S. albogriseolus* showed that strains related to this species were isolated from terrestrial, mangrove, and marine environments [58,59,60]. For strains isolated from sea sediments, it was reported to produce methyl-4,8-dimethylundecanate that can inhibit the growth of fish pathogenic bacteria and be used as an alternative drug for aquaculture management [59]. Moreover, *S. albogriseolus* isolated from marine sediments of Jiaozhou Bay, China, produced new lactone echinosporins with antiproliferative activity against human chronic myelogenous leukemia (K562) (IC_50_ = 91.5 ± 26.5 µM) and human colorectal adenocarcinoma (HCT-15) (IC_50_ = 25.1 ± 1.8 µM) [60]. This demonstrated that *S. albogriseolus* strains isolated in marine environment have the potential to produce a new chemical entity.

The second clade in the phylogenic tree of marine sediment-derived *Streptomyces* from the Visayan Sea was divided into two phyletic lines with only two isolates clustered together. The first branch was isolate DSD017^T^ (1480 bp) that showed a low genetic relatedness to its GenBank match, *S. diastaticus* subsp*. ardesiacus* NBRC 15402^T^ (99.11% similarity), with only a 47% bootstrap value. Strains related to *S. diastaticus* subsp. *ardesiacus* were reported to be isolated from the terrestrial environment [61,62]. A plant endophyte strain of *S. diastaticus* produced a new dimeric phenazine, diastaphenazine, and was described to exhibit weak cytotoxic activity against human large-cell lung carcinoma (H460) (IC_50_ = 14.9 µM), hepatocellular liver carcinoma (HepG2) (IC_50_ = 28.8 µM), colon carcinoma (HCT116) (IC_50_ = 65.2 µM), cervix carcinoma (HeLa) (IC_50_ = 82.5 µM), and gastric carcinoma (BGC-823) cell lines (IC_50_ = >100 µM). Additionally, the compound showed antibacterial activity against *S. aureus* (ATCC 25923) (MIC value = 64 µg/mL); however, it showed no activity against *E. coli* (ATCC 25922) and *C. albicans* (ATCC 10231) at 128 µg/mL [62]. *S. diastaticus* subsp. *ardesiacus* was described to harbor four NRPS gene clusters, one hybrid PKS/NRPS gene cluster, at least four PKS-I gene clusters, two PKS-II, and three PKS-III gene clusters [63].

Another branch was isolate DSD037^T^ with 99.91% similarity to *S. litmocidini* NRRL B-3635^T^ according to the match of its nearly complete 16S rRNA gene sequence (1161 bp). Isolates DSD015^T^, DSD004^T^, DSD006^T^, and DSD011^T^ formed individual clades (clade three to clade six) along with corresponding *Streptomyces* species matched in the GenBank. Isolate DSD015^T^ (1358 bp) showed bootstrap values below 65% indicating a low evolutionary relatedness to its matched species *S. ambofaciens* NBRC 12836^T^ (99.48% similarity). Isolates DSD004^T^ (1339 bp), DSD006^T^ (1479 bp), and DSD011^T^ (1401 bp) showed a close relationship to *S. rubrogriseus* DSM 41477^T^ (99.39% similarity), *S. cacaoi* NBRC 12748^T^ (99.66% similarity), and *S. cellulosae* NBRC 13027^T^ (99.93% similarity), respectively, with bootstrap values ranging from 76% to 100%. These reference strains were isolated from terrestrial soils. For example, most of *S. rubrogriseus* were isolated from soil and its genome harbors four NRPS clusters, one hybrid PKS/NRPS cluster, at least three PKS-I, two PKS-II, and two PKS-III clusters. These biosynthetic gene clusters were predicted to synthesize secondary metabolites such as coelibactin, calcium-dependent antibiotics (CDA), and coelimycin [63]. In the case of *S. cacaoi* NBRC 12748^T^, this strain was isolated from cacao beans in Nigeria [64]. Recently, a new antibacterial peptide, pentaminomycin C, was isolated from the extract of *S. cacaoi* NBRC 12748^T^ that can inhibit Gram-positive bacteria, including *Micrococcus luteus, Bacillus subtilis* and *S. aureus* [65]. A related study on a marine sediment-derived *S. cacaoi* of Mersin Coastline, Turkey, isolated three new polyether-type metabolites that showed antimicrobial activity against vancomycin-resistant *E. faecium*, methicillin-resistant *S. aureus* and *C. albicans,* and cytotoxic activity against numerous cancer cell lines, including HeLa (IC_50_ = 32.6 ± 0.1 µM), CaCo-2 (IC_50_ = 9.4 ± 1.1 µM), PC-3 (IC_50_ = 8.4 ± 1.5 µM), and A549 (IC_50_ = 20.1 ± 0.3 µM) [66]. This report indicates that strains of *S. cacaoi* isolated from a marine environment are capable of synthesizing new chemical entities with biological applications. The *S. cellulosae* NBRC 13027^T^, to which DSD011^T^ is identical, was isolated from garden soil and is a known producer of fungichromin, a macrolide antibiotic used to treat candidiasis [67]. The *Streptomyces* sp. strain DSD011^T^ produces angucycline antibiotics fridamycin A and fridamycin D [12].

The last clade was composed of *Streptomyces* sp. strains DSD012^T^ (1479 bp), DSD035^T^ 1480 bp), DSD036^T^ (1478 bp) and DSD039^T^ (1472 bp) displayed a bootstrap value of 80% to *S. enissocaesilis* NRRL B-16365^T^ (99–100% similarity), suggesting a close evolutionary relatedness of the species. Although strain DSD016^T^ (1360 bp) and its most closely related species, *S. mutabilis* NRRL ISP-5169^T^ (99.78% similarity) were clustered together on clade 7, it formed a subclade showing a low genetic relatedness to each other and supported by its bootstrap value < 65%. Strains related to *S. mutabilis* NRRL ISP-1569^T^ were terrestrial in origin [68,69,70,71]. *Streptomyces* MS-6-6^T^, with a 100% nucleotide gene sequence similarity to *S. mutabilis*, it was isolated from soil samples in Saudi Arabia. This strain produced a polyketide macrolide, treponemycin, that exhibited a promising anti-tuberculosis activity (MIC = 13.3 µg/mL) and extended a wide spectrum of antimicrobial activity against Gram-positive, Gram-negative bacteria, and *C. albicans* [71]. Most of the reported and related strains of *S. enissocaesilis* NRRL B-16365^T^ were isolated from soil samples [72,73,74]. *S. enissocaesilis* that was isolated from a waste discharge soil was able to synthesize silver (Ag), selenium (Se), and zinc oxide (ZnO), natural products with antibacterial activity against Gram-positive and Gram-negative pathogens, including *S. aureus* (MIC = 8.4–16.9 µg/mL), *K. pneumoniae* (MIC = 33.75–67.5 µg/mL), and *P. aeruginosa* (MIC = 8.4–16.9 µg/mL) [74]. Comparing with the previous studies on the terrestrial origin of *Streptomyces* reference strains, this is the first time to recover cultivable closely related *Streptomyces* strains from marine sediments of the Visayan Sea with potential activity against ESKAPE pathogens and ovarian cancer.

### 2.3. Detection of PKS (Types I and II) and NRPS Domains in Streptomyces Isolates

We then investigated the marine sediment-derived *Streptomyces* isolates for their potential to produce secondary metabolites by detecting the presence of biosynthetic gene cluster domains through PCR-based screening targeting polyketide synthase type I (type I PKS) and type II (type II PKS), and for non-ribosomal peptide synthetase (NRPS). The PCR-based approach of amplifying genes for polyketides and non-ribosomal peptides can be an efficient way to detect and predict the type of natural products that can be assembled using PKS and NRPS machinery. A total of six primers were used to amplify the ketosynthase (KS) domain and adenylation domain (AD) in active strains, where two primer sets were designed to target the KS domain of type I PKS (KSMAF-KSMBR; KSF-KSR), three primer sets for type II PKS (KS1F-KS1R; KSα–KSβ; 540F-1100R), and one primer set (A3F-A7R) to detect the adenylation domain for NRPS. The visualization of a strong, unambiguous amplicon band in the agarose gel was the qualifier of PKS genes (Supplemental Figures S2–S5). *Streptomyces* strains isolated in Islas de Gigantes harbor genes for type I PKS amplified using KSMAF and KSMBR primers, except for *Streptomyces* sp. strain DSD011^T^. Despite the absence of type I PKS amplification, *Streptomyces* sp. strain DSD011^T^ harbors type II PKS gene clusters responsible for producing two polycyclic aromatic polyketide angucycline antibiotics, fridamycin A and fridamycin D [12].

*Streptomyces* sp. strain DSD016^T^, with 16S rRNA gene sequences highly similar to *Streptomyces mutabilis*, did not harbor genes for type I PKS amplified using KSF-KSR primers. *Streptomyces mutabilis* was reported to produce two 18-membered macrolides, N-acetylborrelidin B and borrelidin [70], suggesting that, despite the absence of KSF-KSR amplification, *Streptomyces* sp. strain DSD016^T^ still had the machinery to produce PKS type-I compounds. All *Streptomyces* isolates harbor genes for type II PKS amplified using KS1F-KS1R primers (Supplemental Figure S3A**)**, while *Streptomyces* sp. strain DSD006^T^ has no amplification for type II PKS using the 540F-1100R primer set (Supplemental Figure S3C). *Streptomyces* sp. strain DSD006^T^ is closely related based on its 16S rRNA gene sequences to *S. cacaoi*, which was known to produce a macrolide perimycin [75], a glycosylated lantibiotic cacaoidin [76], and an antibacterial cyclic pentapeptide, pentaminomycin C [65].

Interestingly, we observed different band patterns in agarose gel targeting type II PKS using KSα–KSβ primers (Supplemental Figure S3B). The dendrogram of strains amplified using KSα–KSβ revealed eight clusters based on band patterns, including six monophyletic clusters (Supplemental Figure S4). Six strains with ambiguous bands in the agarose gel were considered negative results for the KSα–KSβ primer set: *Streptomyces* sp. strains DSD004^T^, DSD006^T^, DSD016^T^, DSD017^T^, DSD035^T^, and DSD037^T^. However, the band size detected on the *S. cacaoi* strain was not the expected size between 800 and 900 bp. Nevertheless, distinct bands were observed in agarose gel using KSα–KSβ primers. *Streptomyces* sp. strain DSD011^T^ did not harbor genes for type I PKS, indicating that the strain has the machinery specific for type II PKS. The majority of the *Streptomyces* strains harbor genes for NRPS, indicating that these strains can be non-ribosomal peptide producers except for *Streptomyces* sp. strain DSD004^T^ (Supplemental Figure S5). The variations on the amplified genes using PKS and NRPS primer sets suggest the capability of the strains to produce diverse polyketides and non-ribosomal peptides.

### 2.4. Antibiotic Activity against ESKAPE Pathogens

To assess the potential of *Streptomyces* isolates to inhibit the growth of clinically significant pathogens, the isolates were screened against drug-resistant ESKAPE pathogens *E. faecium*, *S. aureus*, *K. pneumoniae*, *A. baumannii*, *P. aeruginosa*, and *E. cloacae*. These bacteria are the leading cause of life-threatening nosocomial infections due to their drug resistance mechanisms [1,77,78]. The biomass of *Streptomyces* isolates was extracted by ethyl acetate (Supplemental Figure S6) and tested against ESKAPE pathogens using a Minimum Inhibitory Concentration (MIC) assay. A 40% hit rate for activity against *S. aureus* (6 isolates—DSD004^T^, DSD006^T^, DSD011^T^, DSD015^T^, DSD016^T^, and DSD037^T^) and *A. baumannii* (6 isolates–DSD006^T^, DSD011^T^, DSD037^T^, DSD041^T^, DSD042^T^, and DSD43^T^) (Table 1) were observed. The least hit rate of 13% (2 isolates—DSD006^T^ and DSD036^T^) was observed against vancomycin-resistant *E. faecium*, a leading cause of hospital acquired infection [79]. Only four *Streptomyces* isolates, i.e., DSD006^T^, DSD011^T^, DSD041^T^, and DSD042^T^, were active against the Gram-negative members of ESKAPE pathogens, namely, *K. pneumoniae, A. baumannii*, *P. aeruginosa*, and *E. cloacae*. The low hit rate could be attributed to the complex outer membrane and efflux pumps possessed by Gram-negative bacteria, which modify drug binding sites/targets and changes in cell permeability resulting in a reduced intracellular drug accumulation to reach the intracellular target [1,77,78]. The crude extract of *Streptomyces* sp. strain DSD006^T^ inhibited the growth of all the ESKAPE pathogens with MIC values ranging from 0.15 mg/mL to 10 mg/mL depending on the target pathogen (Table 1, Supplemental Table S2). Crude extracts of *Streptomyces* sp. strain DSD011^T^ also showed notable antibacterial activity against ESKAPE pathogens with MIC values ranging from 0.312 mg/mL to 20 mg/mL differing on the pathogen it targeted.

The *Streptomyces* sp. strain DSD011^T^ produces angucycline antibiotics that target the growth of *S. aureus*, harboring the *SSCmec* type I gene as a drug-resistant biomarker [12]. The identity of the compounds produced by *Streptomyces* sp. strain DSD011^T^ against *K. pneumoniae*, *A. baumannii*, *P. aeruginosa*, and *E. cloacae* are currently being investigated.

The crude extract of five *Streptomyces* isolates DSD012^T^, DSD017^T^, DSD035^T^, DSD039^T^, and DSD040^T^, are inactive against all ESKAPE pathogens. Interestingly, we observed strain-level antibacterial activity variation among isolates DSD040^T^, DSD041^T^, DSD042^T^, and DSD043^T^ (Table 1, Supplemental Table S2). Based on the 16S rRNA sequence and phylogeny analyses, these isolates belong to the same clade. They are identical to *S. albogriseolus* NBRC 3413^T^ and possess NRPS and type I and II PKS domains (Figure 2). Moreover, these *Streptomyces* strains were isolated from the same ecological site, off the coast of Cabugao Island (Supplemental Figure S1). Isolate DSD040^T^ was inactive against all ESKAPE pathogens; two isolates (DSD041^T^ and DSD042^T^) were active against the four Gram-negative members of ESKAPE pathogens; DSD043^T^ was only active against *A. baumannii* (Table 1). This phenomenon could be explained by the BGC genomic variation and metabolomics diversity among related strains of *Streptomyces,* as reported based on the publicly available genome sequence analysis [80,81,82,83,84]. We also observed the strain-level antibacterial activity variation among marine sediment-derived *S. parvulus* and *S. rochei* (manuscript under review) collected from the different ecological niches. These new findings demonstrate that mining closely related *Streptomyces* species strains from underexplored ecological niches is the future direction for antibiotic drug discovery further study.

### 2.5. Antiproliferative Activity against Ovarian Cancer Cells

**Figure 3:**
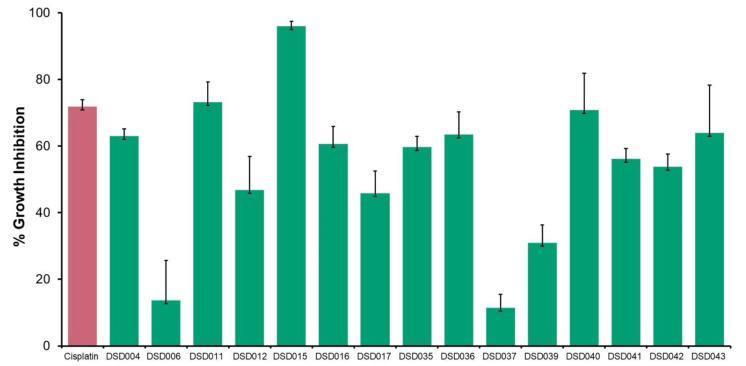
Antiproliferative screening of marine sediment-derived *Streptomyces* crude extracts from Visayan Sea against human ovarian carcinoma, A2780 (ECACC 93112519). Crude extracts (green) were tested at 2 mg/mL, positive control (red) cisplatin was tested at 0.0041 mg/mL.

The antiproliferative activities of 15 crude extracts from marine sediment-derived *Streptomyces* isolates were evaluated using human ovarian carcinoma, A2780 (ECACC 93112519) using MTT Assay. The result showed that 67% of 10 crude extracts inhibited the growth of A2780 cells at a concentration of 2 mg/mL (Figure 3). Isolate DSD015^T^ had the highest inhibitory activity at 96%, followed by DSD011^T^ (73%) and DSD040^T^ with 71% inhibition. The results showed that the marine sediment-derived *Streptomyces* from the Visayan Sea exhibited varying degrees of bioactivity against human ovarian cancer. Interestingly, we observed that isolates exhibiting anticancer activities against human ovarian cancer, DSD015^T^ and DSD040^T^, showed very weak or no antibacterial activities against ESKAPE pathogens, suggesting that the secondary metabolites produced by these isolates are selective at targeting receptors for proliferation in ovarian cancer cells and not receptors found in bacterial cells. The diversity of secondary metabolites produced by the marine sediment-derived *Streptomyces* supports the idea that the Visayan Sea represents an underexplored reservoir of pharmaceutically important streptomycetes.

### 2.6. Bioactivity Correlation with Biosynthetic Gene Clusters

**Figure 4:**
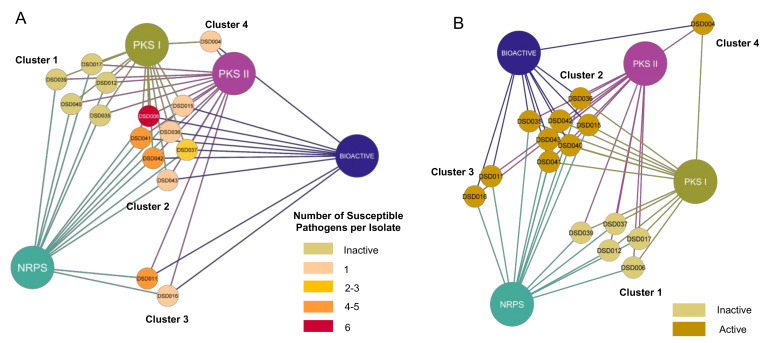
(**A**) Correlation network of the antibacterial capacity of 15 marine sediment-derived *Streptomyces* isolates and their biosynthetic gene clusters (BGCs). The number of susceptible pathogens against each isolate was correlated with the number of BGCs detected in their genome. Big nodes represent the BGCs. The 15 isolates are presented in small nodes with their corresponding isolate code. The color of each node indicates the number of pathogens that were inhibited by each isolate. (**B**) Correlation network of the cytotoxicity of 15 Gigantes isolates and their biosynthetic gene clusters (BGCs). The cytotoxicity against A2780 ovarian carcinoma was correlated with the number of BGCs detected in their genome. Big nodes represent the BGCs. The 15 isolates are presented in small nodes with their corresponding isolate code. The color of each node indicates whether each strain was active (cytotoxic) or inactive.

With the confirmation of multiple BGCs in all 15 *Streptomyces* isolates, we hypothesized that antibiotic activity was positively correlated with the number of BGCs present in its genome. Thus, type I and II PKS, and NRPS were associated with active and inactive *Streptomyces* isolates. Relationships formed were visualized using Cytoscape v3.8.2 (Free Software Foundation, Boston, MA, USA) [85]. The network visualization showed that 15 marine sediment-derived *Streptomyces* isolates aggregated into four major clusters based on their identified BGCs and antibacterial activity, regardless of the number of susceptible pathogens (Figure 4A). We observed that the active and inactive *Streptomyces* isolates were distinctively separated into different groups. Among the ten active strains, seven (47%) isolates had NRPS, type I and type II PKS (Cluster 2), while *Streptomyces* sp. strain DSD011^T^ and DSD016^T^ lacked type I PKS (Cluster 3) and *Streptomyces* sp. strain DSD004^T^ lacked NRPS (Cluster 4). Interestingly, active isolates that harbored all three genes (Cluster 2) expressed different bioactivity strengths. *Streptomyces* sp. strains DSD015^T^, DSD036^T^, and DSD043^T^ were only active against one pathogen. On the contrary, *Streptomyces* sp. strain DSD011^T^ (Cluster 3), which lacked type I PKS, inhibited the growth of *S. aureus* and four Gram-negative pathogens. Furthermore, five strains (*Streptomyces* sp. strains DSD012^T^, DSD017^T^, DSD035^T^, DSD039^T^, and DSD040^T^) (Cluster 4) did not elicit antibacterial activity against all test pathogens despite harboring type I and type II PKS and NRPS in their genome. A similar report described a low correlation between the antibacterial activity of isolates and their detected biosynthetic genes through PCR-based amplification among intertidal marine sediment-derived *Streptomyces* [86].

Similarly, a relationship analysis of 15 marine sediment-derived *Streptomyces* isolates cytotoxicity and BGCs showed clustering of isolates based on the present BGCs and bioactivity (Figure 4B). Active and inactive strains were aggregated into different clusters. However, active strains were further clustered according to the present BGCs in their genome, which revealed that even strains with only one to two BGCs present (Cluster 3 and 4 in Figure 4B) could still inhibit the growth of ovarian cancer cells. Furthermore, all inactive isolates harbored all three BGCs. Both correlation analyses revealed that the number of BGCs present in an isolate does not necessarily dictate its antibacterial and cytotoxic capacity, contrary to our initial hypothesis. The detection of BGCs through PCR does not directly imply the production of secondary metabolites, as the activation and translation of these genes significantly differ in the laboratory setting. Nevertheless, multiple BGCs in active and inactive strains demonstrated their genetic capacity to produce secondary metabolites.

### 2.7. Metabolomics Analysis and Metabolite Profiling of Closely Related Marine Sediment-Derived Streptomyces albogriseolus Strains

**Figure 5:**
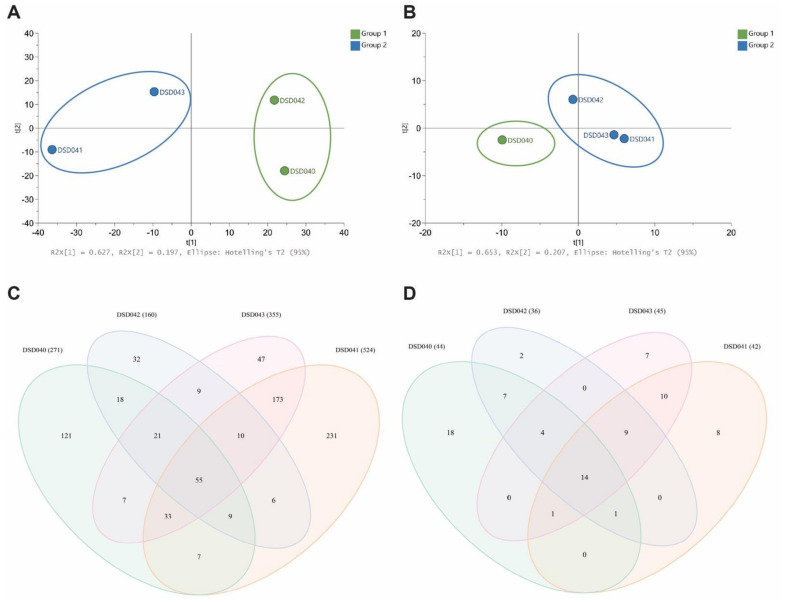
PCA score plots (**A**) Positive Ion Mode and (**B**) Negative Ion Mode of extracts from four marine sediment-derived *Streptomyces albogriseolus* strains DSD040^T^, DSD041^T^, DSD042^T^, and DSD043^T^. Venn analysis (**C**) Positive Ion Mode and (**D**) Negative Ion Mode of metabolites identified from LCMS analysis of four *Streptomyces albogriseolus* strains. Numbers in parentheses show the total number of metabolites per strain.

Motivated by the variation in the biological activities of closely related *Streptomyces* species strains, the metabolite profiles in the extracts of four *S. albogriseolus* strains were analyzed. Cultures of *S. albogriseolus* strains DSD040^T^, DSD041^T^, DSD042^T^, and DSD043^T^ were extracted with EtOAc and analyzed by high-performance liquid chromatography electrospray triple quadrupole mass spectrometry (HPLC-ESI-QQQ-MS). Triplicate profiles were combined for each strain for a comparative multivariate analysis and untargeted metabolite profiling. The principal component analysis (PCA) scores plot showed a variation in the chemical profiles of the four *S. albogriseolus* strains for metabolites detected in positive and negative ion modes. It revealed that taxonomically identical species do not display the same metabolites extracted from their biomass. The scores PC1 62.7% and PC2 19.7% revealed dissimilarities in the metabolome of the four *S. albogriseolus* strains in positive ion mode (Figure 5A). *S. albogriseolus* strains DSD040^T^ and DSD042^T^ were clustered together, while strains DSD041^T^ and DSD043^T^ formed a separate cluster. Extracts from the four *S. albogriseolus* strains showed different numbers of metabolite composition (Figure 5C). A total of 779 metabolite features were identified in the positive ion mode for all four species, in which 55 were shared among the four *S. albogriseolus* strains. These metabolites probably originated from the same BGC as detected in Section 2.3. Interestingly, each strain displays unique metabolites, with strain DSD041^T^ showing the highest number (231) of strain-specific metabolites, while strain DSD042^T^ displayed the least number (32) of strain-specific metabolites. These findings suggest that there are strain-specific BGCs present in *S. albogriseolus* strains. Further, there were metabolites detected in two to three strains, indicating that *S. albogriseolus* strains isolated from the sediments of the Visayan Sea harbor a diverse suite of BGCs. Our results concur with reports showing strain specific BGC mutations observed in *Streptomyces* species, which may drastically affect the organism’s metabolite production or chemodiversity [83,84,87].

A few metabolites were detected in negative ion mode when compared to the positive ion mode. Nonetheless, the *S. albogriseolus* strains displayed a variation in their chemical composition. The PCA plot with PC1 65.3% and PC2 20.7% revealed the clustering of similar strains. Specifically, strains DSD041^T^, DSD042^T^, and DSD043^T^ were clustered together, while DSD040^T^ were separated from these strains as they formed another cluster (Figure 5B). These findings corroborate the reports on metabolomics diversity among related strains of *Streptomyces* based on the publicly available genome sequence analysis [83,84]. To highlight, the type of clustering of metabolites observed in negative ion mode correlates well with the antibiotic activity of these *S. albogriseolus* strains. The strain DSD040^T^ showed no antibacterial activity, while the three strains exhibited antibacterial activities against *A. baumannii*. Based on the Venn diagram of metabolite profiling (Figure 5D), nine metabolites were shared by strains DSD041^T^, DSD042^T^, and DS043^T^. One of these metabolites was responsible for its activity against *A. baumannii*. The metabolome variation observed in this study provided insights into the functional implication on the variation in antibiotic activities of the four *S. albogriseolus* strains. Strain DSD040^T^ had the highest number (18) of unique metabolites, followed by strains DSD041^T^, DSD043^T^, and DSD042^T^. No shared metabolites were found between strains DSD042^T^ and DSD043^T^, strains DSD041^T^ and DSD042^T^, strains DSD043^T^ and DSD040^T^, and strains DSD040^T^ and DSD041^T^. In both positive and negative ion modes, strain DSD042^T^ had the lowest number of metabolites present, 160 and 36, respectively. Additionally, it was observed that in positive and negative ion modes, strains DSD041^T^ and DSD043^T^ shared the highest number of metabolites as compared with other pairs of *S. albogriseolus* strains.

There were antibiotic and anticancer compounds reported from *S. albogriseolus*. An antibiotic methyl-4,8-dimethylundecanate (Supplemental Figure S7A) was isolated from a marine *S. albogriseolus* strain [59]. Additionally, there were anticancer compounds such as alkaloid albogrisins (Supplemental Figure S7B) and eunicellin diterpenoids microeunicellols (Supplemental Figure S7C) reported from *S. albogriseolus* strains [88,89]. We investigated if these compounds were present in the extracts of marine sediment-derived *S. albogriseolus* from the Visayan Sea. The antibiotic methyl-4,8-dimethylundecanate was not detected in the extracts of four *S. albogriseolus* strains; however, microeunicellol A, *m*/*z* 361.19 [M + H]^+^ was detected in all four *S. albogriseolus* strains. The anticancer, albogrisin C *m*/*z* 517.22 [M + H]^+^ was present in strains DSD040^T^, DSD041^T^, and DSD043^T^. The compound was not found in strain DSD042^T^.

Our findings in metabolomics and metabolite profiling offer a new chemical space of untapped secondary metabolites that were potentially biosynthesized by individual *Streptomyces* strains. Hence, strain prioritization based on metabolite profiling should be taken into consideration when developing strategies for natural product discovery.

### 2.8. Antibiotic activity of Streptomyces albogriseolus Strain DSD042^T^ against ESKAPE Pathogens

Based on the above findings on the chemodiversity and variation in antibiotic and anticancer activities within the *S. albogriseolus* strains, a special focus was placed on *S. albogriseolus* strain DSD042^T^ to investigate its extract for antibiotic activity against ESKAPE pathogens. The strain DSD042^T^ was chosen due to its 100% identity to *S. albogriseolus* (Figure 2). Briefly, the strain was cultured for 14 days in a 100 L MM1 solid medium incubated at RT (27–29 °C). First, the agar containing the cells of strain DSD042^T^ was diced and extracted three times with ethyl acetate. Next, the crude extract was concentrated under vacuum and partitioned between double-distilled water and ethyl acetate (1:1, *v*/*v*) to remove primary metabolites and water-soluble molecules in the nutrient medium. Finally, the ethyl acetate extract was concentrated and dried to yield 10.8 g of extracted material from 100 L of solid medium with DSD042^T^ cells.

The extract was dissolved in methanol and subjected to gel permeation chromatography (GPC) using Sephadex LH-20 with methanol as a mobile phase. The GPC yielded 476 fractions of 9 mL each that were then pooled into four major fractions (Supplemental Table S3) according to its UV profile at 254 nm (Supplemental Figure S8). Fractions G2 and G3 contained metabolites with UV peaks at 254 nm, while fractions G1 and G4 did not have UV peaks at 254 nm.

Subsequently, these four pooled GPC fractions were tested for antibiotic activity against the ESKAPE pathogens. The G1 and G2 fractions, which contained the bulk of the crude fraction, were not active against the ESKAPE pathogens (Table 2). However, we observed that fractions G3 and G4, which were eluted towards the later part of chromatography, showed antibacterial activities. Therefore, we inferred that the antibiotic compounds have an inherently small molecular weight. Compared to positive controls, fraction G3 exhibited activities against *S. aureus*, *K. pneumoniae*, *A. baumannii*, *P. aeruginosa*, and *E. cloacae,* but not to *E. faecium*. It showed high activity against *S. aureus* and *E. cloacae* with 12.6 mm ZOI at 5 mg per disc concentration. Its activities against *A. baumannii* and *E. cloacae* were comparable to meropenem at tested concentrations. The last GPC fraction, G4, showed activities to *E. faecium*, *S. aureus*, *K. pneumoniae*, *A. baumannii*, and *E. cloacae,* but not *P. aeruginosa*. The *S. aureus* and *E. cloacae* with 16.6 mm and 13.0 mm ZOI, respectively, showed the most sensitivity to fraction G4. Its activity against *S. aureus* and *K. pneumoniae* was comparable to vancomycin and imipenem, respectively, at the tested concentrations.

The metabolites present in fractions G3 and G4 were evaluated by high performance liquid chromatography with UV and electrospray triple quadrupole mass spectrometry (HPLC-UV-ESI-QQQ-MS) and MS/MS analysis. Eight UV-prominent peaks in positive mode were present in fraction G3. These peaks were *m*/*z* 219.15, 260.15, 321.15, 338.30, 379.40, 381.15, 563.45, and 637.30 [M + H]^+^. Five peaks were detected in negative mode—*m*/*z* 311.20, 325.25, 417.30, 431.30, and 505.35 [M − H]^−^. 

In fraction G4, there were eleven prominent mass peaks detected in positive mode. These were *m*/*z* 336.35, 379.40, 425.25, 505.40, 545.35, 559.45, 585.40, 637.30, 663.45, 685.45, and 726.40 [M + H]^+^. The *m*/*z* 379.40 and 637.50 [M + H]^+^ are peaks that were also detected in fraction G3. There were two prominent mass peaks in negative mode—*m*/*z* 463.35 and 505.40 [M − H]^−^.

MS/MS data from these mass peaks were obtained and analyzed using the online platform the Global Natural Products Social (GNPS) [90]. The MS/MS spectral data returned no match with reference to GNPS, indicating the presence of metabolites with new chemical entities or the absence of similar compounds in the GNPS repository. Untargeted metabolomics is still a big challenge due to the diversity of the chemical composition of metabolites; thus, our findings further emphasized to continue our efforts on targeted isolation, purification, and identification of metabolites against ESKAPE pathogens.

## 3. Materials and Methods

### 3.1. Marine Sediment Selection and Culture Dependent Isolation

Thirty (30) marine sediment samples were collected in March 2016 in Islas de Gigantes group of islands in the Philippine archipelago (11°35′39″ N, 123°20′11″ E) using the method described by Sabido et al. [12]. The samples were air-dried and inoculated in solid medium of ISP4 (International *Streptomyces* Project Medium 4) (Difco) with filtered seawater using the dry stamp method (DSM) and heat shock method (HSM) [11,12,13]. For DSM, a sterile cotton swab was pressed onto the dried sediments and then stamped onto the solid medium in a clockwise direction to create a serial dilution effect while HSM was carried out by adding sterile artificial seawater into the sediments (1:4 *w*/*v*) and heating at 56 °C, shaken vigorously, then 50 µL of the dilution was inoculated into the solid medium. The inoculated plates were incubated at room temperature (25 to 28 °C) for 15 to 30 days.

### 3.2. Salt Tolerance

The salt tolerance test was performed according to the protocol of Sabido et al. [12]. Bacterial strains were isolated and were tested for salt tolerance using MM1 media consisting of 0.2% (*w*/*v*) peptone, 1% (*w*/*v*) starch, 1.8% (*w*/*v*) agar, and 0.4% (*w*/*v*) yeast. Artificial seawater (pH 7.77–7.78) was prepared at different NaCl concentrations (0%, 3%, 5%, 7%, 10%, 12% 15%, and 20%) and was added to the media for the test prior to inoculation. Plated media was divided into four sections to accommodate four isolates in each plate. Each plate was prepared in triplicate. Incubation was at 27 ± 2 °C (room temperature, RT) for 14 days. Substrate and aerial mycelium and diffused pigments were noted at the end of the incubation period.

### 3.3. Phylogenetic Analysis Using Small Subunit 16S rRNA Gene Sequences

Genomic DNA extraction, PCR amplification, and sequencing methods were performed according to procedures adopted in Sabido et al., 2020 [12] (Supplemental Table S4). The 16S rRNA sequences were aligned using CLUSTAL W. The species-level affiliation of the sequences was validated using sequences from the BLAST server from National Center for Biotechnology Information (NCBI) (U.S. National Library of Medicine, Rockville Pike, Bethesda, MD, USA) [91]. The phylogenetic tree was constructed using a multiple alignment with maximum likelihood method in MEGA 7.0 software (Pennsylvania State University, PA, USA) [56] and the resultant tree topologies were evaluated by bootstrap analysis based on 1000 replicates. The evolutionary distance was calculated using the Kimura two-parameter model for nucleotide sequences [92].

### 3.4. PCR Amplification of Secondary Metabolite Biosynthetic Genes

The PCR amplification of secondary metabolite biosynthetic genes was performed according to procedures adopted in Sabido et al., 2020 [12] using the primers designed to detect the conserved regions of ketosynthase (KS) and adenylation (AD) domains necessary for type I PKS, type II PKS, and NRPS biosynthesis (Supplemental Table S5) [10,28,93,94,95]. The occurrence of type I and type II PKS and NRPS was correlated with the antimicrobial activity of the isolates, and the relationship was visualized in a Cytoscape v3.8.2 (Free Software Foundation, Boston, MA, USA) [85] using Inverted Self-organizing Map Algorithm with 1000 iterations.

### 3.5. Cultivation and Extraction of Biomass

The biomass extracts were prepared by growing the *Streptomyces* cells in the marine medium 1 (MM1) agar for 14 days at 25–28 °C according to the published method and protocols [11,12,13]. The biomass was extracted three times with ethyl acetate (1:1 *w*/*v*) and concentrated in vacuo until dried extract was obtained for bioassays and metabolomics analysis.

### 3.6. Determination of Minimum Inhibitory Concentration against ESKAPE Pathogens

ESKAPE pathogens encompass six such pathogens with growing multidrug resistance and virulence: *Enterococcus faecium*, *Staphylococcus aureus*, *Klebsiella pneumoniae*, *Acinetobacter baumannii*, *Pseudomonas aeruginosa* and *Enterobacter* spp. ESKAPE pathogens are responsible for majority of nosocomial infections and can escape the biocidal action of antimicrobial agents [1,77,78]. The ESKAPE (*E. faecium* ATCC 700221, *S. aureus* ATCC BAA-44, *K. pneumoniae* ATCC BAA-1705, *A. baumannii* ATCC BAA-1605, *P. aeruginosa* ATCC BAA-1744 and *E. cloacae* ATCC BAA-2341) pathogens that were used in this study were identified as drug resistant by American Type Culture Collection (ATCC).

The crude extracts of actinomycetes of Islas de Gigantes were prepared using DMSO as solvent, which were then screened for antibacterial activity against ESKAPE pathogens using microbroth susceptibility assay. The minimum inhibitory concentrations of the crude extracts were determined by two-fold serial dilution starting from an initial test concentration of 2.5 mg/mL to 4.8 µg/mL against *E. faecium* ATCC 700221 and *S. aureus* ATCC BAA-44 and 20 mg/mL–39 µg/mL against *K. pneumoniae* ATCC BAA-1705, *A. baumannii* ATCC BAA-1605, *P. aeruginosa* ATCC BAA-1744 and *E. cloacae* ATCC BAA-2341 except for the crude extracts of DSD004^T^ and DSD035^T^ which were tested at 2.5 mg/mL–4.8 µg/mL. Ampicillin (512 µg/mL–1 µg/mL), gentamycin (512 µg/mL–1 µg/mL), and vancomycin (512 µg/mL–1 µg/mL) were used as positive control and DMSO as negative control. The bacterial suspension with an optical density of 1 × 10^6^ CFU/mL at 600 nm was added into the wells and incubated for 18–24 h at 37 °C. The optical density was measured at 600 nm using an absorbance microplate reader (BioTek^TM^ ELx808, BioTek, Winooski, VT, USA). The last concentration showing more than 90% growth inhibition was considered as the MIC_90_ (µg/mL) of the extract [96]. The assay was conducted in triplicates.

### 3.7. Anticancer Assay against Ovarian Cancer Cells

The antiproliferative activity of Gigantes crude extracts was evaluated using the modified method of MTT Assay. Target cell line used was human ovarian carcinoma, A2780 (ECACC 93112519), purchased from Sigma-Aldrich/Merck. The cells were maintained in RPMI-1640 medium (Sigma, St. Louis, MO, USA) supplemented with 10% fetal bovine serum (FBS) and 100 units/mL of penicillin, 100 µg/mL of streptomycin, and incubated in humidified 5% CO_2_ at 37 °C for 24 h. Seeding of cells was conducted in a sterile 96-well plate, in triplicate, at an optimized concentration of 3.5 × 10^5^ cells/mL (35,000 cells/100 µL/well) prior to incubation at 37 °C, 5% CO_2_ and RH = 85–95% for 24 h. The medium was replaced by fresh medium containing different Gigantes crude extracts (2 mg/mL), positive control cisplatin (0.0041 mg/mL) in normal saline solution, and 0.1% DMSO (negative control) in designated wells and incubated for another 24 h using the same parameters. The next day preceded with medium replacement of a fresh medium containing 10 µL of MTT (5 mg/mL) to each well before 4 h incubation at 37 °C, 5% CO_2_ and RH = 85–95%. Medium was carefully removed leaving 10 µL volume and pellets of formazan crystals were solubilized in 100 µL DMSO while pipetting up and down (three times). Microplate reader was set to factor, double orbital at 500 rpm and shaking for 30 s before reading the result at 570 nm using CLARIOstar Multi Mode microplate reader (BMG Labtech GmbH, Offenburg, Germany).

The percent growth inhibition was calculated as follows:

% Cell Viability = ((Absorbance of Treated Cells − Absorbance of Blank)/(Absorbance of Negative Control − Absorbance of Blank)) × 100(1)

% Growth Inhibition = (100% − % Cell Viability)(2)

### 3.8. Gel Permeation Chromatography (GPC)

Approximately ~1.7 g dried crude extract of strain DSD042^T^ was dissolved in 50 mL methanol, filtered and loaded to a 93.98 cm × 19.05 cm column Sephadex^®^ LH-20 (bead size: 27–163 µm, bed volume: 2207 mg/cm^3^, flowrate: 9 cm^3^/min). The column was eluted with methanol and 476 of 9 mL fractions were collected. The UV absorbance of the GPC fractions was measured at 254 and 365 nm using nano spectrophotometer. The eluted fractions were pooled based on the average absorbance value at 254 nm. Three batches of GPC were performed loading a total of 5.1 g. The pooled fractions were concentrated in vacuo using the rotary evaporator and dried further in centrifugal evaporator. The dried samples were stored in −80 °C freezer until used for bioactivity screening and chemical profiling analyses.

### 3.9. Disk Diffusion Assay of Streptomyces albogriseolus Strain DSD042^T^ GPC Fractions

The GPC fractions of *S. albogriseolus* strain DSD042^T^ were subjected to antibacterial activity screening against multidrug-resistant ESKAPE pathogens by disk diffusion assay. The overnight bacterial strains were inoculated into Mueller Hinton Broth (MHB) and optical density of the bacterial was measured at 600 nm using a microplate reader (BioTek ELx808, Winooski, VT, USA). The bacterial density of the test pathogens was adjusted to 1 × 10^6^ CFU/mL and spread evenly into the surface of 0.8% brain heart infusion agar *E. faecium* and 0.8% Mueller Hinton Agar (MHA) plates for *S. aureus*, *K. pneumoniae*, *A. baumannii*, *P. aeruginosa,* and *E. cloacae*. The positive control used was based on the sensitivity of the antibiotics per pathogen. Cefoxitin (30 µg/disk), ciprofloxacin (30 µg/disk), imipenem (10 µg/disk), meropenem (10 µg/disk), and vancomycin (30 µg/disk) were the positive control used and methanol as negative control. The GPC fractions of *S. albogriseolus* DSD042^T^ (5 mg/ disk) was dispensed into 6 mm circular disks and impregnated into agar plates with pathogen along with the controls. The assay was conducted in triplicates. The plates were incubated for 18–24 h before measuring zone of inhibition.

### 3.10. High-Pressure Liquid Chromatography Mass Spectrometry (HPLCMS), Multivariate and Venn Analyses of the Crude Extracts of Streptomyces Isolates from the Visayan Sea

High-Pressure Liquid Chromatography Mass Spectrometry analysis was performed on a Shimadzu^TM^ LCMS-TQ 8045 instrument equipped with a Prominence HPLC system (Shimadzu Corporation, Kyoto, Japan) (SIL-20A HT autosampler, LC-20AD pump system, SPD-20AV UV Vis Detector) using Phenomenex Synergi^TM^ reversed-phase column (particle size: 4 µm, diameter: 100.0 × 2.0 mm) in gradient elution system LCMS grade water with 0.1% formic acid (solvent A) to 100 % LCMS grade acetonitrile with 0.1% formic acid, ACN (solvent B). The dried extracts were dissolved in LCMS grade methanol and filtered using 0.2 µm syringe filter. The sample loaded was 10 µL of 1 mg/mL of the extracts (strains DSD040^T^, DSD041^T^, DSD042^T^, and DSD043^T^). The instrument parameters for the elution were performed in a solvent flow rate of 0.3 mL/min and with oven temperature at 40 °C, starting with 60% solvent A at 0.18 min, gradually increasing to 100% solvent B at 25 min to 30 min, then back to 60% solvent A at 34 min to 35 min. Data acquisition was stopped at 35 min. The block temperature was 400 °C, and DL temperature was 250 °C. The nebulizing gas flow rate was 2.0 L/min nitrogen while the drying gas flow rate was 10.0 L/min nitrogen. The event time was 0.2 s at Q3 scan positive mode and Q3 scan negative mode. The masses were analyzed in both positive and negative ion modes with range *m*/*z* 200–1500. LabSolutions^TM^ software (v5.89, Shimadzu Corporation, Kyoto, Japan) was used for control and data processing.

For the metabolomics analysis, Profiling Solutions Software^TM^ (v1.1, Shimadzu Corporation, Kyoto, Japan) was used to generate the heat map showing significant intensities of ions (*m*/*z*) obtained from positive and negative ion modes LCMS-TQ experiment with tolerance of 500 mDA, retention time tolerance of 1.0 min, and intensity threshold of at least 1 million (1 × 10^6^) counts. Peak integration algorithm set to Chromatopac max peak of one (1), width of 10 sec, slope of 3000 per min, drift 75,000 per min, T. DBL of 1000 min, smoothing of 5 counts. The normalized data of mass peaks and signals with relative standard deviation (RSD) of <10 was exported to multivariate analysis software (SIMCA 16.0.1 Software^TM^ Sartorius Stedim Data Analytics AB, Umeå, Sweden) to generate the principal component analysis score and loading plots using Pareto scaling. Venn diagram analyses of the metabolites processed in Profiling Solutions™ (v1.1 Shimadzu Corporation, Kyoto, Japan) were created using the Venn Diagram package in RStudio statistical program v1.2.5042 (RStudio, Boston, MA, USA).

### 3.11. High-Pressure Liquid Chromatography Mass Spectrometry (HPLCMS) and MS/MS Analysis of GPC Fractions and Dereplication

High-Pressure Liquid Chromatography Mass Spectrometry analysis was performed on a Shimadzu^TM^ LCMS-TQ 8045 instrument equipped with a Prominence HPLC system (Shimadzu Corporation, Kyoto, Japan) (SIL-20A HT autosampler, LC-20AD pump system, SPD-20AV UV–VIS Detector) using Phenomenex Synergi^TM^ reversed-phase column (particle size: 4 µm, diameter: 100.0 × 2.0 mm) in a gradient elution system from 80% LCMS grade water with 0.1% formic acid (solvent A) to 100% LCMS grade acetonitrile, ACN (solvent B) with 0.1% formic acid. The dried GPC fractions were dissolved in LCMS grade methanol. The sample was load at 10 µL at 0.8 mg/mL for G3 and 20 µL at 0.5 mg/mL for G4. The instrument parameters for the elution were performed in a solvent flow rate of 0.3 mL/min and with oven temperature at 40 °C, starting with 80% solvent A at 0.01 to 2.0 min, gradually increasing to 100% solvent B at 7 min to 17 min, then back to 80% solvent A at 19 min to 20 min. Data acquisition was stopped at 20 min. The block temperature was 400 °C, and DL temperature was 250 °C. The nebulizing gas flow rate was 2.0 L/min nitrogen while the drying gas flow rate was 10.0 L/min nitrogen. The event time was 0.2 s at Q3 scan positive mode and Q3 scan negative mode. The masses were analyzed in both positive and negative ion modes with range *m*/*z* 200–1500. LabSolutions software (v5.89, Shimadzu Corporation, Kyoto, Japan) was used for control and data processing.

MS/MS experiments were performed on the peaks of interest at 10 V to 70 V collision energy. The identity of the metabolites was obtained after comparison with a library search online workflow of GNPS through MASST Search Tool (University of California, San Diego, CA, USA) [60]. A single MS/MS spectrum was matched against all public spectral libraries that included peptides, lipids, small molecules, and pharmacologically active natural products. The spectral match was required to have a parent mass tolerance of 2.0 Da, ion tolerance of 0.5 Da, score threshold of 0.70, and at least 4 matched peaks and minimum cosine score of 0.70.

### 3.12. Statistical Analysis

The results of the disk diffusion assay were analyzed by Paired *t*-Test Analysis using GraphPad Prism software (v9.2, GraphPad Software, LLC, San Diego, CA, USA). Raw datasets of zone of inhibition (ZOI) in each GPC fraction were plotted to the software and run to determine the significant difference between the means of two groups.

## 4. Conclusions

In conclusion, the unexplored marine sediments of the Visayan Sea were an abode to antibiotic and anticancer-producing actinomycetes. This study demonstrated that bioprospecting on these unexplored marine sediments led to the isolation and identification of 15 halotolerant actinomycetes with a high genetic similarity to *Streptomyces* species. These isolates harbor BGC domains (types I and II PKS and NRPS). The majority were shown to synthesize bioactive secondary metabolites that target ESKAPE pathogens and human ovarian carcinoma. In-depth analyses on the closely related *Streptomyces* species strains revealed divergence in the metabolites produced, resulting in the variation in the bioactivities. Probing on the semi-purified extracts enabled to separate and characterize the bioactive compounds to non-bioactive, in this case through size exclusion. The findings of this study support the idea that the marine sediments of the Visayan Sea represent an untapped reservoir of actinomycetes which can serve as a source of new antibiotics and anticancer leads. Importantly, strains with similar phylogenetic classification should not be discarded in the screening process for bioprospection. Further, series of purification and biological and chemical profiling partnered with metabolomics analyses and genomic mining will be the future directions of this study.

## Figures and Tables

**Table 1:** Antibiotic activity screening of the extracts of marine sediment-derived *Streptomyces* from Visayan Sea against ESKAPE pathogens.

Isolate Code	GenBank Accession Number	Nearest Related Strain	% Identity	Antibacterial Activity * (MIC_90_)					
				*E. faecium*	*S. aureus*	*K. pneumoniae*	*A. baumannii*	*P. aeruginosa*	*E. cloacae*
DSD004^T^	MZ323891	*Streptomyces rubrogriseus* DSM 41477^T^	99.39						
DSD006^T^	MZ323976	*Streptomyces cacaoi* NBRC 12748^T^	99.66						
DSD011^T^	MT820508	*Streptomyces cellulosae* NBRC 13027^T^	99.93						
DSD012^T^	MZ323983	*Streptomyces enissocaesilis* NRLL B-16365^T^	99.93						
DSD015^T^	MZ323974	*Streptomyces ambofaciens* NBRC 12836^T^	99.48						
DSD016^T^	MZ323997	*Streptomyces mutabilis* NRRL ISP-1569^T^	99.78						
DSD017^T^	MZ336041	Streptomyces diastaticus ** NBRC 15402^T^	99.11						
DSD035^T^	MZ323985	*Streptomyces enissocaesilis* NRLL B-16365^T^	99.86						
DSD036^T^	MZ323984	*Streptomyces enissocaesilis* NRLL B-16365^T^	100.00						
DSD037^T^	MZ323989	*Streptomyces litmocidini* NRLL B-3635^T^	99.91						
DSD039^T^	MZ323987	*Streptomyces enissocaesilis* NRLL B-16365^T^	99.73						
DSD040^T^	MZ323957	*Streptomyces albogriseolus* NBRC 3413^T^	99.86						
DSD041^T^	MZ336042	*Streptomyces albogriseolus* NBRC 3413^T^	99.93						
DSD042^T^	MZ323972	*Streptomyces albogriseolus* NBRC 3413^T^	100.00						
DSD043^T^	MZ323973	*Streptomyces albogriseolus* NBRC 3413^T^	99.93						
% Hit rate				13	40	33	40	26	26

Green: active; Red: non-active; T: type strain; * The ESKAPE pathogens used in this study were drug-resistant *E. faecium* ATCC 700221, *S. aureus* ATCC BAA-44, *K. pneumoniae* ATCC BAA-1705, *A. baumannii* ATCC BAA-1605, *P. aeruginosa* ATCC BAA-1744, and *E. cloacae* ATCC BAA-2341. The activity was scored based on the Minimum Inhibitory Concentration (MIC) of the extracts. ** *S. diastaticus* subsp. *ardesiacus.*

**Table 2:** Antibacterial Activity of *S. albogriseolus* strain DSD042^T^ Gel Permeation Chromatography fractions.

Treatment Code	Disk Content (mg)	Zone of Inhibition (mm)					
		*E. faecium* ATCC 70021	*S. aureus* ATCC BAA-44	*K. pneumoniae* ATCC BAA-1705	*A. baumannii* ATCC BAA-1605	*P. aeruginosa* ATCC BAA-1744	*E. cloacae * ATCC BAA-2341
G1	5.000	-	-	-	-	-	-
G2	5.000	-	-	-	-	-	-
G3	5.000	-	12.6 ± 2.0	9.6 ± 0.5	11.0 ± 1.7 ^c^	9.3 ± 0.5 ^b^	12.6 ± 2.0 ^c^
G4	5.000	11.0 ± 1.0 ^a^	16.6 ± 1.0 ^c^	11.3 ± 0.5 ^c^	10.6 ± 0.5	-	13.0 ± 1.7
Cefoxitin	0.030			13.0 ± 1.7		-	
Ciprofloxacin	0.030				-		-
Imipenem	0.010			11.3^+^ ± 2.0 ^c^	-	16.0^+^ ± 1.7 ^b^	-
Meropenem	0.010			16.0 ± 1.7	9.3 ± 0.5 ^c^		15.0^+^ ± 1.0 ^c^
Vancomycin	0.030	22.0 ± 1 ^a^	16.1 ± 1 ^c^				

Note: Cefoxitin was only tested against *K. pneumoniae* and *P. aeruginosa*; Ciprofloxacin was only tested against *A. baumannii* and *E. cloacae*; Imipenem was only tested against *K. pneumoniae*, *A. baumannii*, *P. aeruginosa*, and *E. cloacae*; Meropenem was only tested against *K. pneumoniae*, *A. baumannii,* and *E. cloacae*; Vancomycin was only tested against *E. faecium* and *S. aureus.*-no activity; + colonies were observed within the zone; ^a^ very statistically significant; ^b^ statistically significant; ^c^ not statistically significant.

## Data Availability

The 16S rRNA gene sequences in this study were deposited in GenBank.

## Supplementary Materials

The following are available online at https://www.mdpi.com/article/10.3390/md19080441/s1, Table S1: Growth of isolates in different NaCl concentrations, Table S2: Minimum inhibitory values concentration of *Streptomyces* crude extracts against ESKAPE pathogens, Table S3: Gel Permeation Chromatography (GPC) Purification of DSD042^T^ crude extract showing four major fractions, color profile, and yield and antibiotic activity against ESKAPE pathogens, Table S4: Primers for amplification of 16S rRNA Gene, Table S5: Degenerate primers for PCR screening of polyketide synthase and non-ribosomal synthetase, Figure S1: Map of the collection sites (shown in red) of marine sediments in the Islas de Gigantes group of islands, Figure S2: Agarose gel electrophoresis of the PKS-I amplicons of *Streptomyces* sp. strains, Figure S3: Agarose gel electrophoresis of the PKS-II amplicons of *Streptomyces* sp. strains, Figure S4: Agarose gel electrophoresis of the NRPS amplicons of *Streptomyces* sp. strains. A3F and A7R were the primers used to amplify the adenylation domain of NRPS, Figure S5: Dendrogram of Amplified Gene Fragments in *Streptomyces* isolates using KSα–KSβ primer set, Figure S6: The 15 DSD isolate dried extracts of Islas de Gigantes extracted using ethyl acetate, Figure S7: Molecular structure of compounds produces by *Streptomyces* strains related to *S. albogriseolus*, Figure S8: Average absorbance of DSD042^T^ GPC fraction showing peaks in G2 and G3 at UV λ_max_ 254 nm.
