# Supplementary material for: Insights into the Variation in Bioactivities of Closely Related Streptomyces Strains from Marine Sediments of the Visayan Sea against ESKAPE and Ovarian Cancer
Source: Mar Drugs. 2021 Jul 31;19(8):441. doi: 10.3390/md19080441 (PMC8399204; doi:10.3390/md19080441)
Supplement: Supplementary file 1 [file marinedrugs-19-00441-s001.zip › marinedrugs-1296569-supplementary.pdf]

Insights into the Variation in Bioactivities of Closely Related *Streptomyces* Strains from Marine Sediments of the Visayan Sea against ESKAPE and Ovarian Cancer

Edna M. Sabido[1], Chuckcris P. Tenebro[2], Dana Joanne Von L. Trono[2], Carmela Vannette B. Vicera[2], Sheeny Fane L. Leonida[1], Jose Jeffrey Wayne B. Maybay[1], Rikka Reyes-Salarda[2,3], Diana S. Amago[1], Angelica Marie V. Aguadera[1], May C. Octaviano[1], Jonel P. Saludes[1,4,5,6]* and Doralyn S. Dalisay[2,3,5,6]*

[1] Center for Natural Drug Discovery and Development (CND3), University of San Agustin, Iloilo City 5000, Philippines; ednasabido@usa.edu.ph (E.M.S.); sfleonida@usa.edu.ph (S.F.L.L.); jjmaybay@usa.edu.ph (J.J.W.B.M.); damago@usa.edu.ph (D.S.A.); aaguadera@usa.edu.ph (A.M.V.A.); moctaviano@usa.edu.ph (M.C.O.)

[2] Center for Chemical Biology and Biotechnology (C2B2), University of San Agustin, Iloilo City 5000, Philippines; ctenebro@usa.edu.ph (C.P.T.); djvtrono@usa.edu.ph (D.J.V.L.T.), cvicera@usa.edu.ph (C.V.B.V.)

[3] Department of Biology, College of Liberal Arts, Sciences, and Education, University of San Agustin, Iloilo City 5000, Philippines; rikkareyes@usa.edu.ph (R.R.S.)

[4] Department of Chemistry, College of Liberal Arts, Sciences, and Education, University of San Agustin, Iloilo City 5000, Philippines; jpsaludes@usa.edu.ph (J.P.S.)

[5] Tuklas Lunas Development Center, University of San Agustin, Iloilo City 5000, Philippines

[6] Balik Scientist Program, Philippine Council for Health Research and Development (PCHRD), Department of Science and Technology, Bicutan, Taguig City 1631, Philippines

* Correspondence: jsaludes@usa.edu.ph (J.P.S); ddalisay@usa.edu.ph (D.S.D.) ; Tel.: +63 33 501-0350

Supplemental Information

Table S1. Growth of *Streptomyces* isolates in different NaCl concentrations.

Isolates		Observations					
		0%	3%	5%	7%	10%	12%
DSD004^T	Growth	+++	+++	+++	++	+	+
	Mycelium Description	mass of gray spores	mass of gray spores	mass of gray spores	no spores	no spores, yellow brown substrate	no spores, yellow brown substrate
	Diffused Pigment	reddish brown	reddish brown	reddish brown	reddish brown	none	none
DSD006^T	Growth	+++	+++	+++	+++	++	+
	Mycelium Description	mass of white spores	mass of white spores	mass of white spores	mass of white spores	mass of white spores	few white spores, light yellow brown substrate
	Diffused Pigment	none	none	none	none	none	none
DSD011^T	Growth	+++	+++	+++	++	-	-
	Mycelium Description	mass of grayish spores	mass of grayish spores	mass of grayish spores	no spores, yellow brown substrate		
	Diffused Pigment	none	none	none	none		
DSD012^T	Growth	+++	+++	+++	++	-	-
	Mycelium Description	mass of grayish and white spores	white and gray spores, yellow brown substrate	white and gray spores, yellow brown substrate	no spores, yellow brown substrate		
	Diffused Pigment	none	none	none	none		
DSD015^T	Growth	+++	+++	+++	++	+	-
	Mycelium Description	mass of gray spores	white and gray spores, yellow brown substrate	white and gray spores, yellow brown substrate	no spores, yellow brown substrate	no spores, yellow brown substrate	
	Diffused Pigment	none	*blue violet	*blue violet	none	none	
DSD016^T	Growth	+++	+++	+++	++	++	-
	Mycelium Description	mass of grayand white spores	gray and white spores, yellow brown substrate	gray and white spores, yellow brown substrate	no spores, yellow brown substrate	no spores, yellow brown substrate	

Isolates		Observations					
		0%	3%	5%	7%	10%	12%
	Diffused Pigment	none	none	none	none	none	
DSD017[T]	Growth	+++	+++	+++	++	+	-
	Mycelium Description	mass of gray spores	mass of gray and white spores	mass of gray spores	no spores, yellow brown substrate	no spores, yellow brown substrate	
	Diffused Pigment	none	red orange	red orange	yellow orange	none	
DSD035[T]	Growth	+++	+++	+++	+++	++	-
	Mycelium Description	mass of gray spores	mass of gray spores	gray and white spores	white spores, yellow brown substrate	no spores, yellow brown substrate	
	Diffused Pigment	none	none	yellow	yellow	light yellow	
DSD036[T]	Growth	+++	++	++	++	-	-
	Mycelium Description	mass of gray spores	no spores, brown substrate	white spores, brown substrate	no spores, brown substrate		
	Diffused Pigment	none	none	none	yellow		
DSD037[T]	Growth	+++	+++	+++	+++	-	-
	Mycelium Description	mass of gray and white spores	mass of white spores	mass of white spores	mass of white spores		
	Diffused Pigment	none	none	brown	none		
DSD039[T]	Growth	+++	++	+++	+++	++	-
	Mycelium Description	mass of gray spores	white spores	white spores, yellow brown substrate	white spores, yellow brown substrate	no spores, yellow brown wrinkled substrate	
	Diffused Pigment	none	none	yellow	yellow	yellow	
DSD040[T]	Growth	+++	+++	+++	++	-	-
	Mycelium Description	mass of gray and white spores	mass of gray and white spores	mass of gray and white spores	mass of gray and white spores		
	Diffused Pigment	none	none	none	none		
DSD041[T]	Growth	+++	+++	+++	++	-	-

Isolates		Observations					
		0%	3%	5%	7%	10%	12%
	Mycelium Description	mass of gray and white spores	mass of gray and white spores	mass of gray and white spores	mass of gray and white spores		
	Diffused Pigment	none	none	none	none		
DSD042T	Growth	+++	+++	+++	++	-	-
	Mycelium Description	mass of gray and white spores	mass of gray and white spores	mass of gray spores	mass of gray spores		
	Diffused Pigment	none	none	none	none		
DSD043T	Growth	+++	+++	+++	++	-	-
	Mycelium Description	mass of gray and white spores	mass of gray and white spores	mass of gray spores	mass of gray and white spores		
	Diffused Pigment	none	none	none	none		

*Selected plates. The growth is indicated in the following condition: good (+++), moderate (++), poor (+), and no growth (-). The presence of mycelium and diffused pigments were also described in each isolate. No growth beyond 12% NaCl concentration.

Table S2. Minimum Inhibitory Concentrations of *Streptomyces* crude extracts against ESKAPE pathogens.

Treatment/Isolates code	MIC$_{90}$ (mg/mL)					
	E. faecium **ATCC 700221**	***S. aureus*** **ATCC BAA-44**	***K. pneumoniae*** **ATCC BAA-1705**	***A. baumannii*** **ATCC BAA-1605**	***P. aeruginosa*** **ATCC BAA-1744**	***E. cloacae*** **ATCC BAA-2341**
DSD004T	>2.5	0.625	>2.5	>2.5	>2.5	>2.5
DSD006T	2.5	0.312	10	10	10	10
DSD011T	>2.5	0.312	20	10	10	10
DSD012T	>2.5	>2.5	>20	>20	>20	>20
DSD015T	>2.5	2.5	>20	>20	>20	>20
DSD016T	>2.5	0.625	>20	>20	>20	>20
DSD017T	>2.5	>2.5	>20	>20	>20	>20
DSD035T	>2.5	>2.5	>2.5	>2.5	>2.5	>2.5
DSD036T	2.5	>2.5	>20	>20	>20	>20
DSD037T	1.25	2.5	20	10	>20	>20

Treatment/Isolates code	MIC90 (mg/mL) *E. faecium* ATCC 700221	*S. aureus* ATCC BAA-44	*K. pneumoniae* ATCC BAA-1705	*A. baumannii* ATCC BAA-1605	*P. aeruginosa* ATCC BAA-1744	*E. cloacae* ATCC BAA-2341
DSD039T	>2.5	>2.5	>20	>20	>20	>20
DSD040T	>2.5	>2.5	20	>20	>20	>20
DSD041T	>2.5	>2.5	20	20	20	20
DSD042T	>2.5	>2.5	20	10	10	20
DSD043T	>2.5	>2.5	>20	20	>20	>20
Ampicillin						>0.512
Gentamicin				0.128	0.001	
Tetracycline	0.001	0.016	0.001	0.032	0.008	0.008
Vancomycin	>0.512	0.002	0.512			

Note: Ampicillin was only tested against *E. cloacae*; Gentamicin was only tested against *A. baumannii* and *P. aeruginosa*; Vancomycin was only tested against *E. faecium, S. aureus* and *K. pneumoniae.* "T" – type strain

Table S3. Gel Permeation Chromatography (GPC) Purification of DSD042 T crude extract showing four major fractions, color profile, and yield.

Sample/Fraction	Fraction	Visible Color Profile	Yield (mg)
**G1**	1-65	Dark brown	1,437.2
**G2**	66-95	Reddish Brown	2,561.31
**G3**	96-150	Dark Brown	851.92
**G4**	151-470	Brownish Yellow	325.09

Table S4. Primers for amplification of 16S rRNA Gene.

Gene	Primers	Primer Sequence (5′ to 3′)
16S rRNA	27F	AGAGTTTGATCCTGGCTCAG
	1492R	TACGGCTACCTTGTTACGACTT
	518F	CCAGCAGCCGCGGTAATACG
	800R	TACCAGGGTATCTAATCC

Table S5. Degenerate Primers for PCR Screening of Polyketide Synthase and Non-Ribosomal Synthase (References: 10, 28, 93-95).

Target Gene for Amplification	Length (bp)	Primer Name	Primer Sequence (5′ to 3′)
PKS-I β-ketoacyl synthase (KS) domains fragments	670	KS-F<br>KS-R	CCS CAG SAG CGC STS YTS CTS GA<br>GTS CCS GTS CCG TGS GYS TCS A
PKS-I KS Domain Fragments	700	KSMAF<br>KSMBR	TSG CSA TGG ACC CSC AGC AG<br>CCS GTS CCG TGS GCC TCS AC
PKS-II KSα and KSβ domain fragments	800-900	KSα<br>KSβ	TSG RCT ACR TCA ACG GSC ACG G<br>TAC SAG TCS WTC GCC TGG TTC
PKS-II KSα domain fragments	613	KS1-F<br>KS1-R	TSG CST GCT TGG AYG CSA TC<br>TGG AAN CCG CCG AAB CCT CT
PKS-II at 5′ portion of KSα genes	554	540F<br>1100R	GGI TGC ACS TCI GGI MTS GAC<br>CCG ATS GCI CCS AGI GAG TG
NRPS Adenylation domain (AD) fragments	700	A3F<br>A7R	GCS TAC SYS ATS TAC ACS TCS GG<br>SAS GTC VCC SGT SCG GTA S

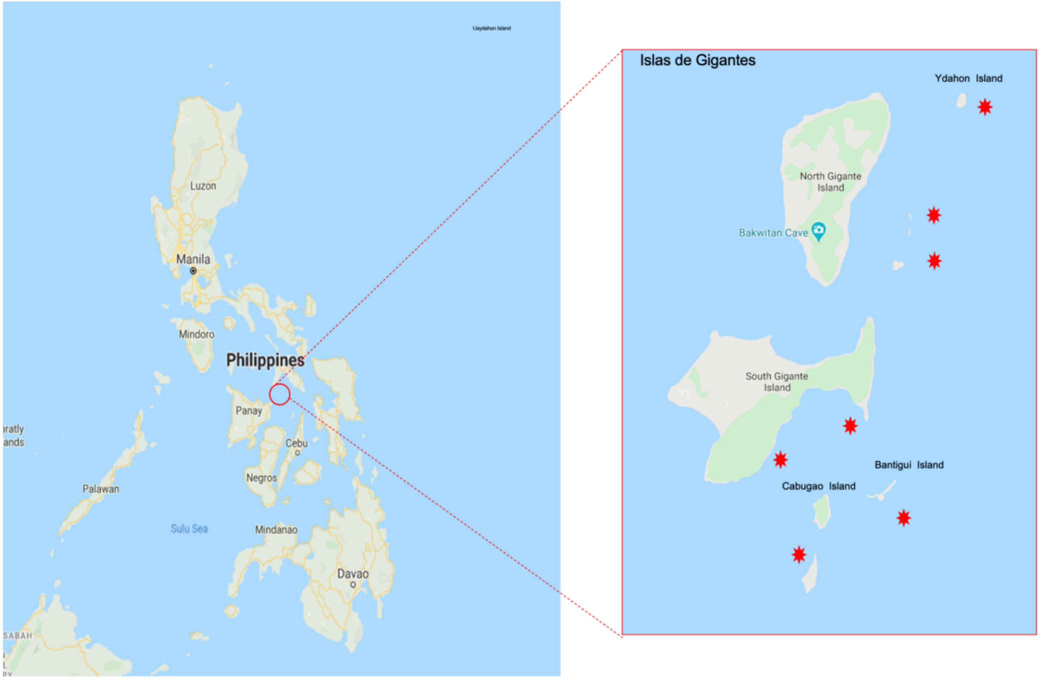

Figure S1. Map of the collection sites (shown in red) of marine sediments in the Islas de Gigantes group of islands.

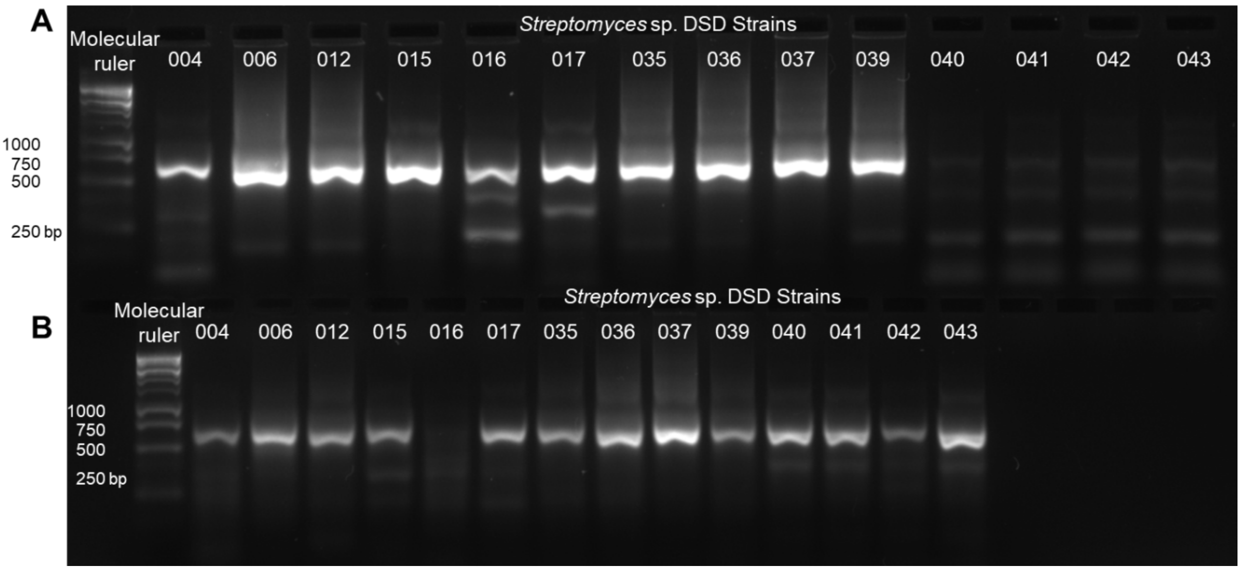

Figure S2. Agarose gel electrophoresis of the PKS-I amplicons of *Streptomyces* sp. strains. Two sets of primers were used to amplify the β-ketoacyl synthetase domain of PKS-I. Fragments of 600-700 bp of PKS-I amplified by A) KSMAF and KSMBR; and B) KSF and KSR primers.

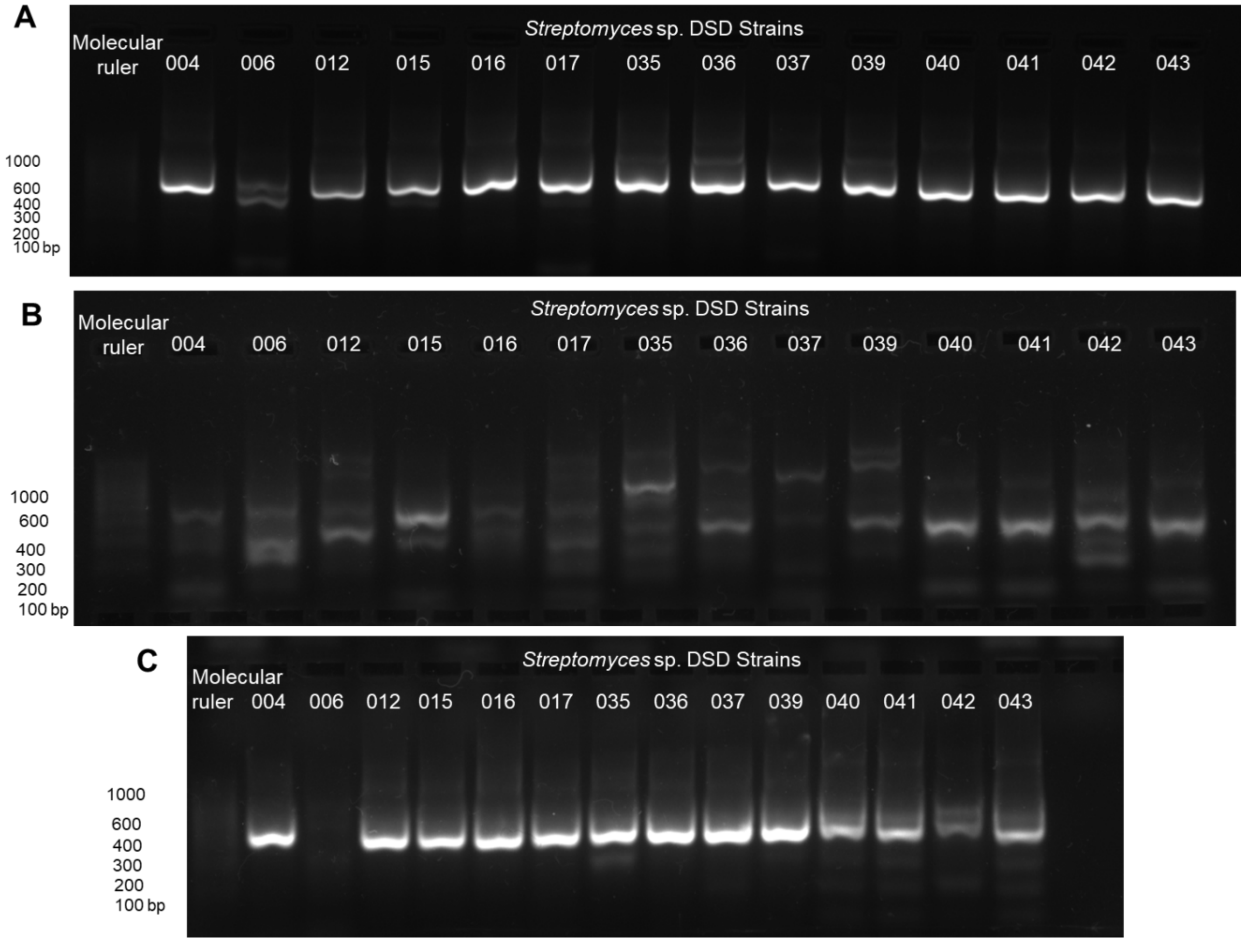

Figure S3. Agarose gel electrophoresis of the PKS-II amplicons of *Streptomyces* sp. strains**.** Three sets of primers were used to amplify the β-ketoacyl synthetase domain of PKS-II. A) KS1F and KS1R primers; B) KSα and KSβ; and C) 540F and 1100R.

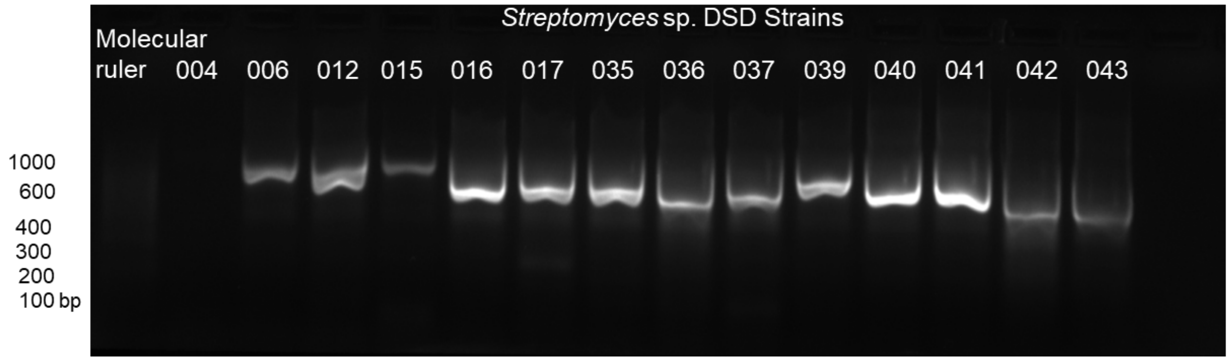

Figure S4. Agarose gel electrophoresis of the NRPS amplicons of *Streptomyces* sp. strains. A3F and A7R were the primers used to amplify the adenylation domain of NRPS.

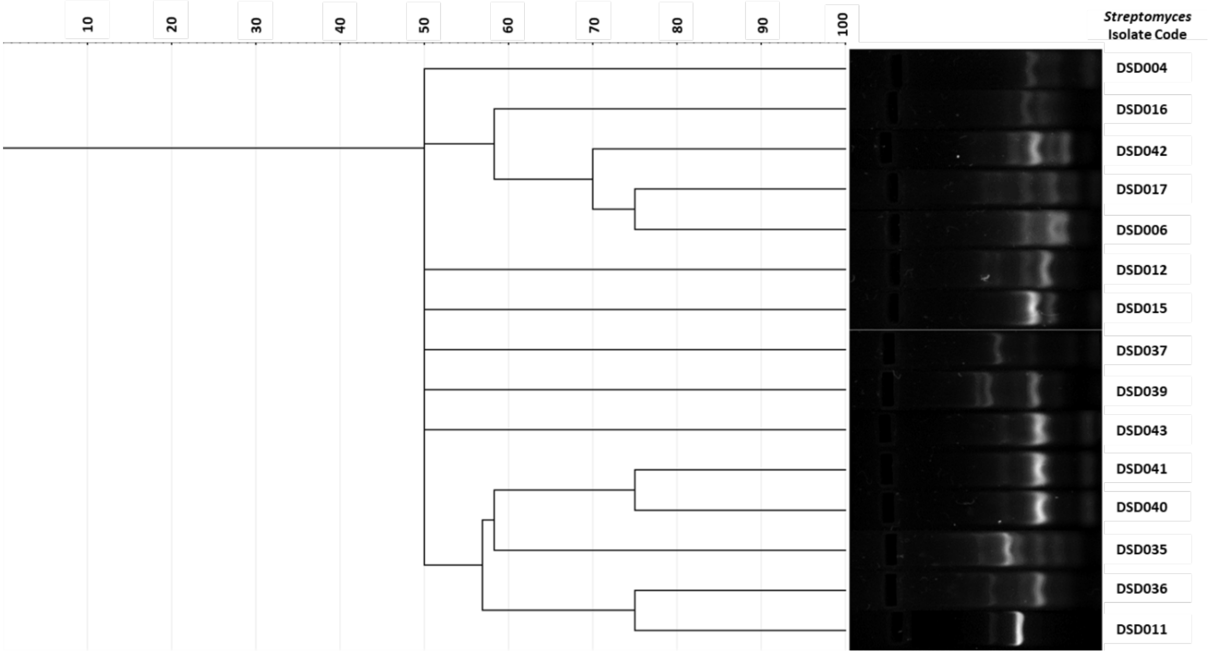

Figure S5. Dendrogram of Amplified Gene Fragments in *Streptomyces* isolates using KSα-KSβ primer set. The UPGMA clustering produced two main groups and eight clusters using the Dice coefficient. Band patterns observed may indicate the diverse aromatic compounds produced by the strains using PKS type-II machinery. The dendrogram was analyzed and generated using the Java program GelJ (Heras, J., Domínguez, C., Mata, E. *et al*. GelJ – a tool for analyzing DNA fingerprint gel images. *BMC Bioinformatics* **2015**, *16*, 270).

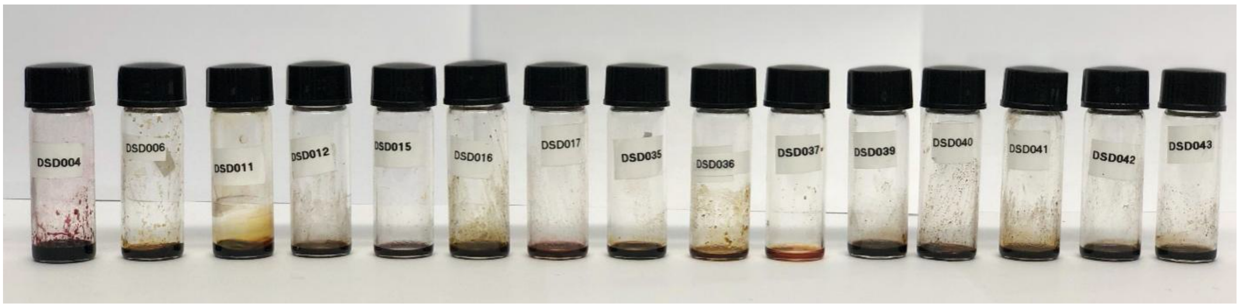

Figure S6. The 15 DSD isolate dried extracts of Islas de Gigantes extracted using ethyl acetate.

A.

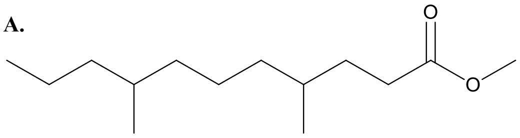

Methyl-4,8-dimethylundecanate [60]

B.

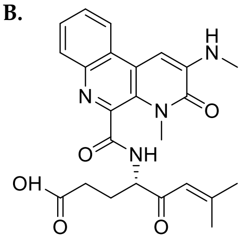

Albogrisin A [91]

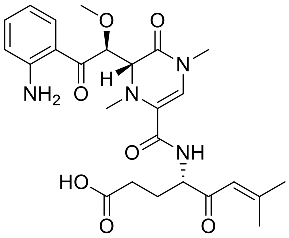

Albogrisin B [91]

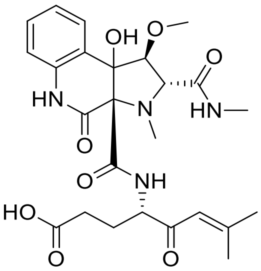

Albogrisin C [91]

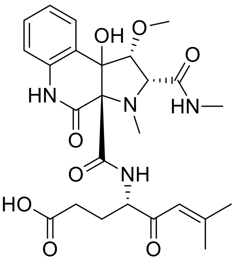

Albogrisin C′ [91]

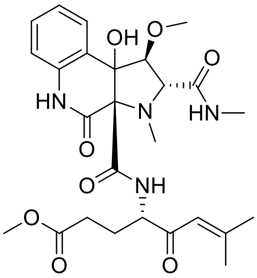

Albogrisin D [91]

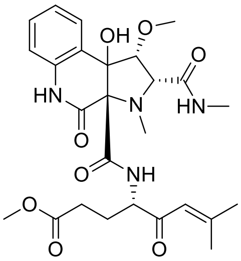

Albogrisin D′ [91]

C.

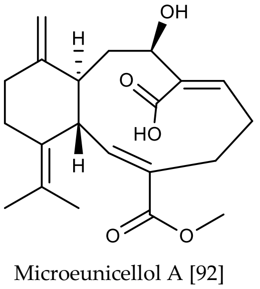

Microeunicellol A [92]

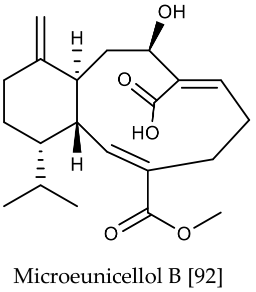

Microeunicellol B [92]

Figure S7. Molecular structure of compounds produced by *Streptomyces* strain related to *S. albogriseolus* (Reference: 88 and 89)

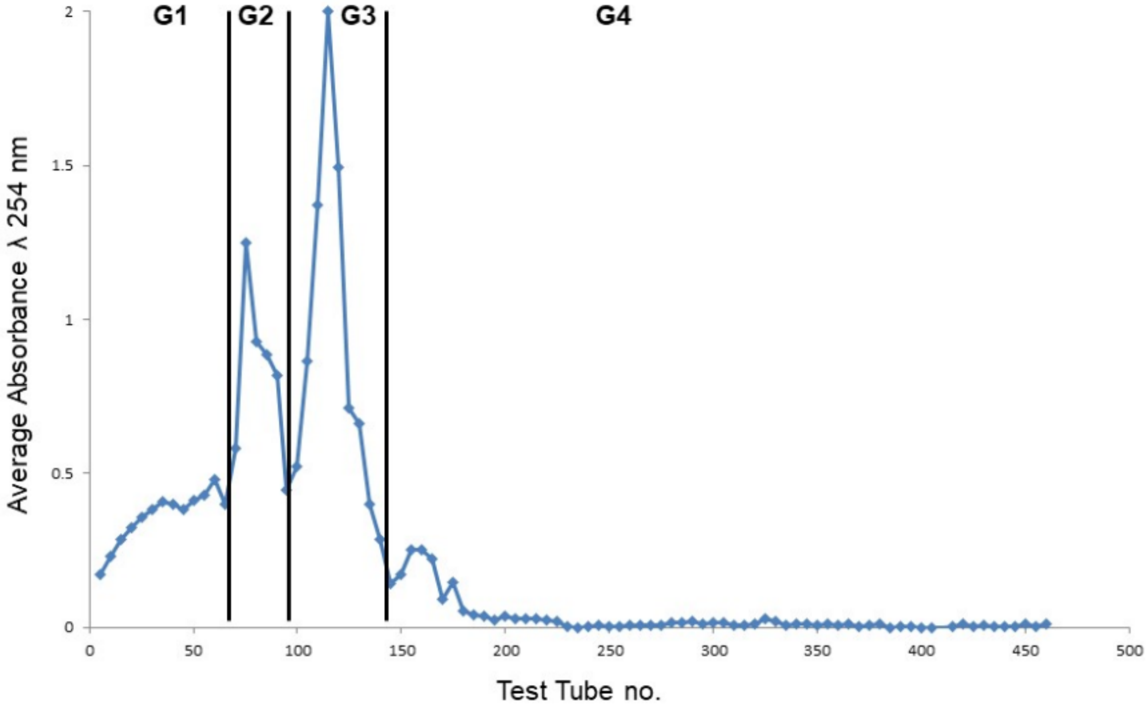

Figure S8. Average Absorbance of DSD042[T] GPC fraction showing peaks in G2 and G3 at UV λ_{max} 254 nm.
